# GABAergic signaling in alcohol use disorder and withdrawal: pathological involvement and therapeutic potential

**DOI:** 10.3389/fncir.2023.1218737

**Published:** 2023-10-20

**Authors:** Ravinder Naik Dharavath, Celeste Pina-Leblanc, Victor M. Tang, Matthew E. Sloan, Yuliya S. Nikolova, Peter Pangarov, Anthony C. Ruocco, Kevin Shield, Daphne Voineskos, Daniel M. Blumberger, Isabelle Boileau, Nikki Bozinoff, Philip Gerretsen, Erica Vieira, Osnat C. Melamed, Etienne Sibille, Lena C. Quilty, Thomas D. Prevot

**Affiliations:** ^1^Campbell Family Mental Health Research Institute of CAMH, Toronto, ON, Canada; ^2^Department of Pharmacology and Toxicology, University of Toronto, Toronto, ON, Canada; ^3^Department of Psychiatry, University of Toronto, Toronto, ON, Canada; ^4^Addiction Division, CAMH, Toronto, ON, Canada; ^5^Division of Neurosciences and Clinical Translation, Department of Psychiatry, University of Toronto, Toronto, ON, Canada; ^6^Department of Psychological Clinical Science, University of Toronto Scarborough, Toronto, ON, Canada; ^7^Institute of Medical Science, University of Toronto, Toronto, ON, Canada; ^8^Institute of Mental Health Policy Research, CAMH, Toronto, ON, Canada; ^9^Temerty Centre for Therapeutic Brain Intervention, CAMH, Toronto, ON, Canada; ^10^Department of Psychology, University of Toronto Scarborough, Toronto, ON, Canada; ^11^Brain Health Imaging Centre, CAMH, Toronto, ON, Canada; ^12^Department of Family and Community Medicine, University of Toronto, Toronto, ON, Canada

**Keywords:** alcohol use disorders, clinical trials, GABA, integrative approach, interventions, pharmacotherapy, translational, unmet need

## Abstract

Alcohol is one of the most widely used substances. Alcohol use accounts for 5.1% of the global disease burden, contributes substantially to societal and economic costs, and leads to approximately 3 million global deaths yearly. Alcohol use disorder (AUD) includes various drinking behavior patterns that lead to short-term or long-lasting effects on health. Ethanol, the main psychoactive molecule acting in alcoholic beverages, directly impacts the GABAergic system, contributing to GABAergic dysregulations that vary depending on the intensity and duration of alcohol consumption. A small number of interventions have been developed that target the GABAergic system, but there are promising future therapeutic avenues to explore. This review provides an overview of the impact of alcohol on the GABAergic system, the current interventions available for AUD that target the GABAergic system, and the novel interventions being explored that in the future could be included among first-line therapies for the treatment of AUD.

## 1. Introduction

Alcoholic beverages have been consumed for recreational purposes in most parts of the world since before recorded history began. According to the latest World Health Organization (WHO) global estimates (WHO, 2021), about 5.1% of the global adult population is living with alcohol use disorders (AUD). Another study by the global burden of disease (GBD) collaborative network reported a 1.5% global AUD prevalence in 2019, highlighting variabilities between countries (Castaldelli-Maia and Bhugra, 2022). Ethanol, the main active component of alcoholic beverages, is currently one of the most used psychoactive drugs on the market. Ethanol produces a state of anxiolysis and disinhibition, which is commonly sought after in social situations or in individuals with AUD (Gilman et al., 2008). Alcohol consumption is also causally related to the development of approximately 230 diseases or disorders, including infectious diseases, malignant neoplasms, cardiovascular system due to ethanol’s effect on blood pressure and inflammation (Chiva-Blanch and Badimon, 2019), mental and behavioral disorders, neurological diseases, digestive diseases, and injuries (Rehm et al., 2017). While consumption patterns vary, the impact of ethanol at low doses on overall health remains unclear (Larsson et al., 2020; Zhao et al., 2023). A recent systematic meta-analysis of cohort studies showed no statistically significant protective effect of alcohol on all-cause mortality at low ethanol intakes (Zhao et al., 2023). Studies have highlighted that abstinence from alcohol has many health benefits, including improved sleep. On the contrary, the risk of certain types of cancer, heart disease, and stroke increases with increased alcohol consumption (Savin et al., 2018; Paradis et al., 2022), and chronic consumption of ethanol in high doses is also linked to feelings of dysphoria, cognitive deficits, and an increased risk of developing AUD (Trantham-Davidson and Chandler, 2015).

Two major diagnostic classification systems are used to define AUD. The Diagnostic and Statistical Manual of Mental Disorders, Fifth Edition (DSM-5), developed by the American Psychiatric Association, defines AUD as a cluster of behavioral and physical symptoms, including withdrawal, tolerance, and craving (American Psychological Association, 2013). The International Classification of Diseases 11^th^ Revision (ICD-11), developed by the World Health Organization, divides AUD into a harmful pattern of alcohol use and alcohol dependence. Alcohol dependence is characterized by “a strong internal drive to use alcohol, which is manifested by an impaired ability to control use, increasing priority given to use over other activities, and persistence of use despite harm or negative consequences” (WHO-ICD11, 2022). According to the ICD-10 definition of AUD, it was estimated that in 2016, approximately 8.6% of adult men and 1.7% of adult women suffered from AUD globally (Carvalho et al., 2019).

AUD may be characterized by the development of tolerance due to homeostatic adaptation in the brain compulsive seeking and withdrawal upon cessation of consumption (Liang and Olsen, 2014). AUD symptomatology includes a wide range of behaviors such as poor control over drinking and impulsivity (a failure to inhibit excessive drive), reward deficiency (a reduced response to natural rewards), maladaptive learning (the growing incentive salience of a drug’s predictive cues with chronic use), the emergence of opponent processes (the power of negative motivational states underlying withdrawal), faulty decision making (inaccurate computation in preparation for action) or automaticity of responses (inflexibility of stimulus–response habits) (Volkow et al., 2013). Due to neuronal dependency on alcohol for regular activity in individuals with AUD, cessation of alcohol consumption often leads to withdrawal (Littleton, 1998). Sudden cessation might result in acute withdrawal symptoms, including delirium, seizures, and cognitive dysfunctions (Jesse et al., 2017; Laniepce et al., 2020). However, the symptoms seen in alcohol withdrawal range in severity depending on the volume and duration of ethanol consumption and inter-individual variability (Newman et al., 2023). Withdrawal symptoms are often related to hyperexcitability, such as insomnia, anxiety, palpitations, agitation, and even seizures (Saunders et al., 2019), likely related to alteration in the functioning of the brain inhibition system.

Due to its hydrophilic nature, ethanol readily penetrates all biological membranes and crosses the blood–brain barrier. Once in the organism, ethanol metabolism happens in the liver but also in the brain due to the presence of alcohol dehydrogenase (ADH), catalase, and P450 (CYP2E1) in both organs. Such metabolism routes produce mainly three metabolites: acetaldehyde, salsolinol, and acetate (Gil-Mohapel et al., 2019; Wilson and Matschinsky, 2020). After reaching the brain, ethanol and its metabolites induce diverse disturbances such as reduced glucose uptake, increased monocarboxylate uptake, dopaminergic, GABAergic, and glutamatergic alterations (Peana et al., 2017).

Since, ethanol and its metabolites act on multiple biological pathways of the central nervous system (CNS), therapeutic interventions relying on various approaches have been developed with variable degrees of efficacy. However, there is still a significant need to understand better the underlying mechanism leading to AUD and associated symptoms and develop more efficient intervention strategies. While impacting many CNS pathways, one of the main pathways altered by alcohol is the inhibitory pathway utilizing gamma-aminobutyric acid (GABA).

This review provides an overview of the impact of ethanol on brain functions related to GABA, describes existing therapeutic interventions, lists their shortcomings, and summarizes the existing knowledge around GABAergic functions in AUD involved in the expression of symptoms and outcomes before providing insight into the development of future therapeutic interventions acting on the GABAergic system.

## 2. Impact of ethanol on brain

**Figure 1:**
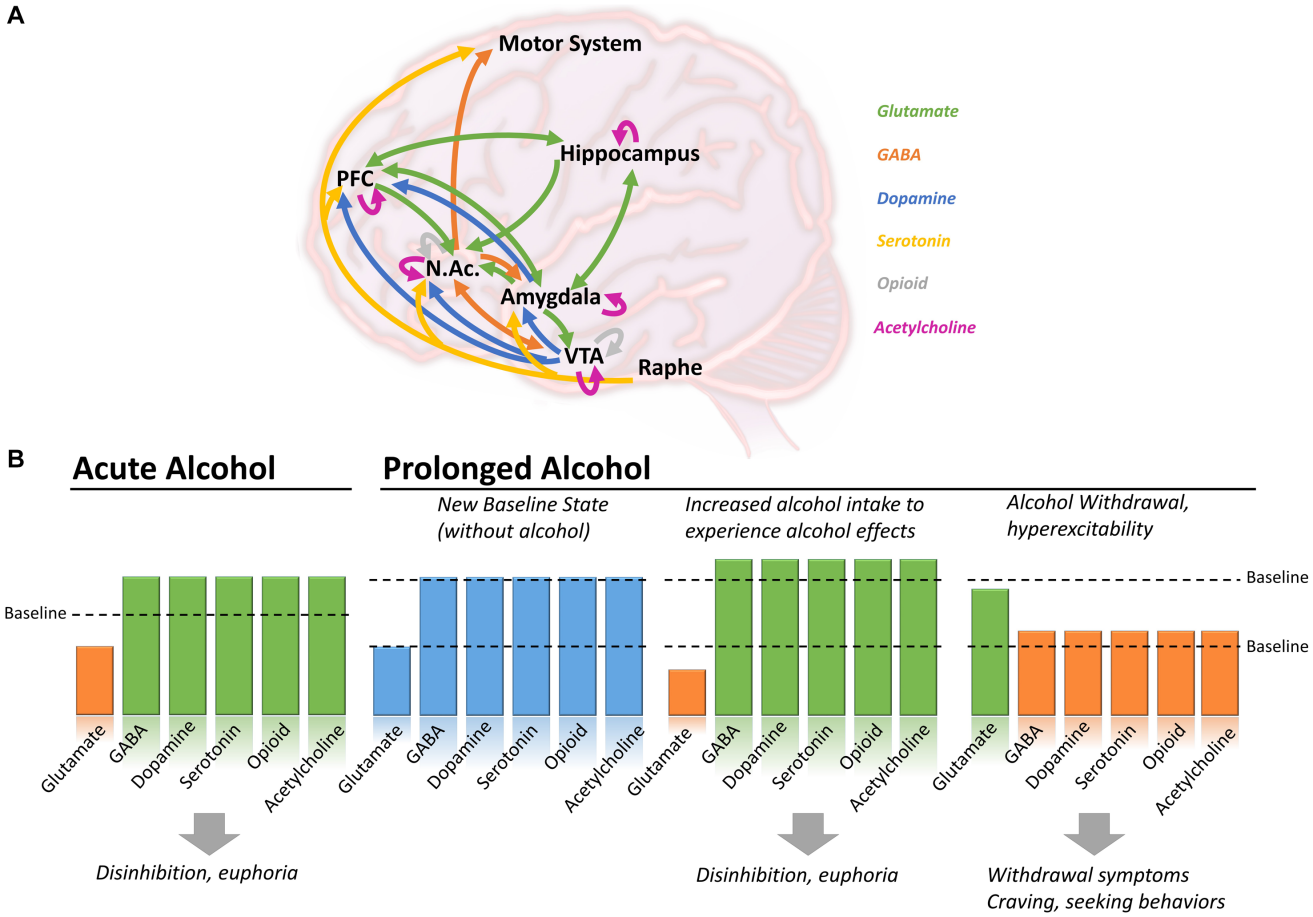
Brain circuits affected by alcohol consumption in the context of acute or prolonged exposure. **(A)** Alcohol induces changes in neutrotransmitters including glutamate (green), GABA (orange), dopamine (blue), serotonin (yellow), opioid (grey) and acetylcholine (purple), in various brain regions. **(B)** During acute alcohol consumption, ethanol induces a decrease in glutamatergic activity and an increased of GABAergic, dopaminergic, serotoninergic, opioid and cholinergic systems. With prolonged alcohol consumption, the different systems establish themselves at a new baseline level. To experience the effect of alcohol, individuals have to further increase their consumption leading to disinhibition and euphoria, but increasing the risk of AUD and dependence. During withdrawal, glutamatergic activity increases above the newly-set baseline, while GABAergic, dopaminergic, serotoninergic, opioid and cholinergic activity decrease, causing withdrawal symptoms, craving and seeking behaviors. Arrows in panel A shows direction of the projections between brain regions. NAc, Nucleus accumbens; PFC, Prefrontal cortex; VTA, Ventral tegmental area.

Ethanol produces a wide variety of behavioral and physiological effects in the body, but exactly how it acts to produce these effects is still poorly understood. Like most dependence-producing substances, ethanol binds and acts on multiple proteins, receptors, and signaling pathways throughout the brain (Figure 1A), including amino acids, opioids, enzymes, and ion channels (Heinz et al., 2009; Koob and Volkow, 2016). The primary targets behind ethanol-induced behavioral phenotypes (disinhibition, hyperlocomotion, and anxiolysis) are GABA_A_ receptors. Besides modulating GABA_A_ receptor activity, ethanol can directly bind and modulate the activity of several proteins, including ionotropic glutamatergic (NMDA) receptors, alcohol dehydrogenase (ADH), and glycine receptors (Grant and Lovinger, 2018). Further, it has been observed that ethanol is capable of indirect modulation of other neurotransmitters (dopamine, serotonin, opioid, and cholinergic), particularly in brain regions involved in the mesolimbic reward system [i.e., amygdala, hippocampus, striatum, and ventral tegmental area (VTA)] via GABAergic/glutamatergic neurons or their respective receptors present on other types of neurons (Abrahao et al., 2017). Therefore, chronic ethanol consumption in large volumes drives a chemical imbalance in the brain and forces a homeostatic response to maintain neurochemical equilibrium and functionality (De Witte, 2004). As the brain chemically adapts to excess ethanol, it forms a new equilibrium in which ethanol becomes integral in neuronal function (Figure 1B; Valenzuela, 1997; Pérez-Ramírez et al., 2022). In individuals with AUD, this is manifested through increased tolerance to the effects of ethanol, which can lead to the consumption of alcohol near toxicity levels to experience the effects of alcohol, such as relaxation, anxiolysis, or disinhibition. Consistent with this notion, magnetic resonance spectroscopy (MRS) studies generally demonstrate lower cortical GABA levels in individuals with AUD, specifically during withdrawal, than in control participants (Prisciandaro et al., 2019; Kirkland et al., 2022; Shyu et al., 2022).

Therefore, the main activity of ethanol is thought to be on glutamatergic and GABAergic signaling pathways, with an increase or decrease of function depending on the state (acute consumption, chronic consumption, or withdrawal), inducing a cascade of events acting on dopamine, serotonin, and endogenous opioid release (Ferraguti et al., 2015).

### 2.1. Impact of ethanol on glutamate and GABA

Preclinical and clinical studies showed that ethanol binds to and inhibits the functions of the glutamatergic receptors (NMDA, AMPA, Kainate, and mGluR5) (Möykkynen and Korpi, 2012; Ferraguti et al., 2015). It also binds to and facilitates the functions of the GABA_A_ and GABA_B_ receptors (Valenzuela and Jotty, 2015; Olsen and Liang, 2017), which, combined with the effect of glutamatergic receptors, causes an overall imbalance in neuronal activity, thought to be responsible for “blackout” moments after acute heavy drinking (Wetherill and Fromme, 2016; Yang et al., 2022) and contributing to excitotoxicity and loss of synaptic plasticity (Chandrasekar, 2013). Data from studies using human transcranial magnetic stimulation (TMS), a non-invasive neuromodulation approach that probes GABA-receptor-mediated cortical inhibition, confirmed that alcohol intake increases GABA-inhibitory neurotransmission and decreases NMDA-receptor-activated excitatory neurotransmission (Ziemann et al., 2015). Interestingly, the activity of ethanol metabolites on glutamatergic and GABAergic targets seems different, which could explain the dynamic changes happening during drinking episodes (see Section 2.6 below).

Preclinical studies in rats have also confirmed the critical impact of ethanol on the regulation of ethanol-maintained responses through GABA_A_ receptor-dependent signaling in the central nucleus of the amygdala (Avegno et al., 2018; Barchiesi et al., 2021; Kisby et al., 2021). Preclinical studies have also confirmed the impact of alcohol on behavioral outcomes [compulsive behavior (Giuliano et al., 2018), withdrawal-induced hyperalgesia (Avegno et al., 2018), increased anxiety (Barchiesi et al., 2021), altered cognitive functions], and biological pathways [GABA and glutamine (McCunn et al., 2022), glutamate (Vengeliene et al., 2005; Mira et al., 2019), dopamine (Ma and Zhu, 2014; Solanki et al., 2020)] as well as provided insights onto therapeutic interventions (Foo et al., 2019).

### 2.2. Impact of ethanol on acetylcholine

Ethanol intake in rats was also shown to bind to the nicotinic-subtype receptor of acetylcholine (Davis and de Fiebre, 2006) and to increase acetylcholine levels in the VTA (Larsson et al., 2005), facilitating the influx of dopamine onto the nucleus accumbens (NAc). Such activity in the VTA and NAc is thought to contribute to positive reinforcement of alcohol. In contrast, modulation of the nicotinic receptors of the hippocampus and amygdala is thought to be involved in negative effects (Tarren et al., 2016). Ethanol’s binding and activity at nicotinic receptors are also thought to interfere with nicotine-induced desensitization, which could explain the high prevalence of co-use of alcohol and tobacco (Davis and de Fiebre, 2006; Addolorato et al., 2012).

### 2.3. Impact of ethanol on dopamine

As a downstream effect of alcohol consumption, ethanol induces an indirect increase in dopamine release and acetylcholine activity from the VTA to the NAc, a brain region strongly associated with reward and motivation (Boehm II et al., 2004). Preclinical Studies have also shown that dopamine is released in the ventral striatum and NAc, contributing to drug reward, which could be further increased by nicotine co-administration (Tizabi et al., 2007). The activation of central GABAergic neurotransmission, particularly through GABA_B_ receptors, is also linked to the mesolimbic dopaminergic neurotransmission during rewarding processes, altogether contributing to the addictive properties of ethanol (Addolorato et al., 2012).

### 2.4. Impact of ethanol on serotonin

Acute alcohol consumption increases serotonin release, contributing to the rewarding aspect of consuming alcohol (Banerjee, 2014). Previous studies showed that acute ethanol augments the firing rate of the serotoninergic 5-HT_3_ receptors, and longer consumption can affect the expression and function of various other subtypes, including 5-HT_2_, without a clear understanding of whether it is a direct effect or mediated by a cascade of events or adaptation (Lovinger, 1997).

### 2.5. Impact of ethanol on opioids

Consumption of alcoholic beverages has also been shown to increase the levels of endogenous opioids (Mitchell et al., 2012), which are subsequently drastically reduced during withdrawal, leading to craving and increasing the risk of opioid-seeking behaviors (Turton et al., 2020). The activity of ethanol at GABA_A_ receptors in the VTA and NAc facilitates endogenous opioid release in the VTA, contributing to the alcohol-induced feeling of euphoria (Colasanti et al., 2012). Opioid-targeting treatments such as naltrexone or nalmefene diminish these effects of alcohol (Turton et al., 2020), providing further evidence of the impact of alcohol on the opioid system.

### 2.6. Impact of ethanol metabolism on various neurotransmitters

Acetaldehyde, salsolinol, and acetate, metabolites of ethanol, seem to participate in the effect of alcohol, but their contribution is less understood. Acetaldehyde in the brain causes euphoria at low doses and plays a vital role in ethanol’s reinforcing properties, thereby facilitating alcohol addiction (Quertemont et al., 2005; Peana et al., 2017). One of the primary studies reported that acetaldehyde increased GABA uptake but did not affect both its release and synthesis (Bobrova and Covaltchuk, 1980). Acetaldehyde has been shown to stimulate dopaminergic neurons (Melis et al., 2007) and μ opioid receptors (Sanchez-Catalan et al., 2014). Acetaldehyde is a highly reactive and short-lived metabolite of ethanol that reacts with biogenic amines like dopamine and forms condensation products like Salsolinol.

Studies reported that salsolinol may exert some of the effects of ethanol by activating μ opioid receptors on GABAergic neurons signaling onto dopaminergic neurons in the mesolimbic system. However, the mechanisms are complex, and it seems like salsolinol would reduce GABAergic activity while ethanol increases it, suggesting opposite responses on GABAergic receptor activity from ethanol and one of its metabolites, also causing a downstream opposite effect on dopamine release (Peana et al., 2017).

Finally, the direct role of acetate on GABAergic regulation has not been reported. However, acetate was reported to contribute to increased cerebral blood flow (Tanabe et al., 2019), increased neuronal excitability, and enhanced glutamatergic activity (Chapp et al., 2021), whereas ethanol boosts GABA-mediated inhibition. Accordingly, existing literature indicates that concrete experimental evidence is required to confirm the effects of ethanol’s metabolites on the GABAergic system.

## 3. GABAergic mechanisms involved in AUD

GABA is the main inhibitory neurotransmitter in the brain. It exerts its function by binding to two types of receptors: GABA_A_ and GABA_B_. GABA_A_ receptors are ionotropic chloride channels (Enna, 2007), while GABA_B_ are metabotropic G-coupled protein receptors (GPCR) (Pinard et al., 2010). GABA_B_ receptors mediate slow inhibitory transmission, while GABA_A_ mediates fast inhibition. GABA_A_ and GABA_B_ have been extensively reviewed for their potential in pharmacotherapies (Sarasa et al., 2020) and link to AUD (Ghit et al., 2021; Holtyn and Weerts, 2022).

**Figure 2:**
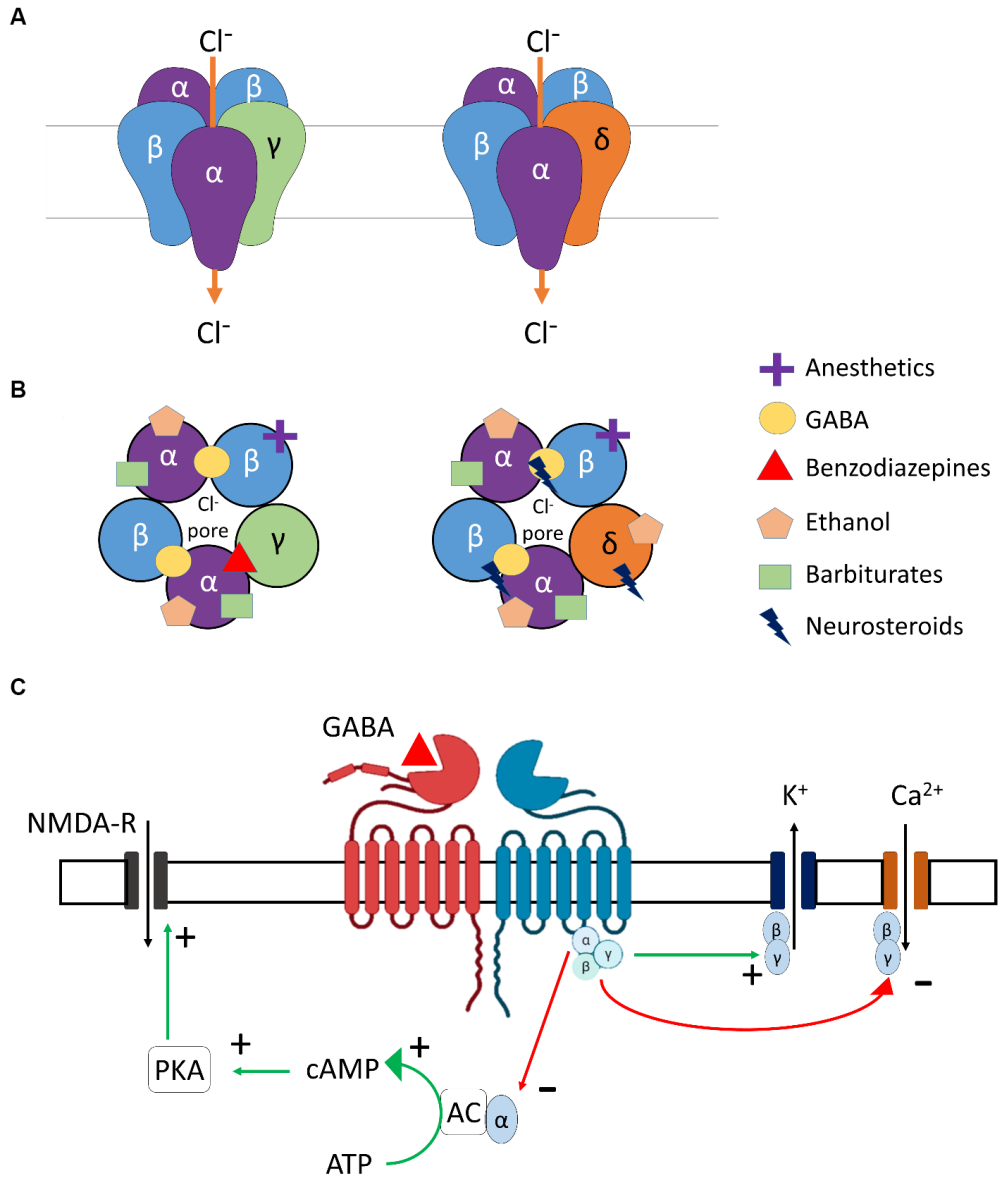
GABA receptor subtypes involved in modulation of ethanol. **(A)** Molecular structures of γ- and δ- subunit-containing GABA_A_ receptors. **(B)** Ligand-specific binding sites of γ- and δ- subunit-containing GABA_A_ receptors. **(C)** Molecular structure and downstream signaling of GABA_B_ receptors. α, β, γ, δ, subunits of GABA_A_ receptor; Cl-, Chloride ion; GABA, Gamma-aminobutyric acid; NMDA-R, N-Methyl-D-aspartic acid receptor; K^+^, Potassium ion; Ca^+2^, Calcium ion; PKA, Protein kinase A; cAMP, Cyclic Adenosine monophosphate; ATP, Adenosine triphosphate; AC, Adenylyl cyclase.

GABA_A_ receptors are heteropentamers composed of various subunits such as α, β, γ, δ, ε, θ, π, and ρ (Figures 2A,B), which are found throughout the brain (Fritschy and Mohler, 1995), including regions involved in alcohol-related use such as the prefrontal cortex, thalamus, cerebellum, or the amygdala (Bowery et al., 1987). Ethanol acts as a positive allosteric modulator (PAM) of GABA_A_ receptors, binding to several subunits, mostly α-subunits, thus explaining its sedative and neuromodulating properties (Ghit et al., 2021). Other PAMs include benzodiazepines and Z-drugs that promote sedation, anxiolysis, muscle relaxation, and anti-seizure properties.

GABA_B_ receptors are the only metabotropic G protein-coupled receptors for GABA (Figure 2C) and can be found in presynaptic (auto-inhibitory) and postsynaptic membranes and distributed throughout the CNS and PNS. The two main subunits of the GABA_B_ receptor are GABA_B_R1 and GABA_B_R2. For the GABA_B_ receptors to be active and functional, these subunits need to interact to form a stable heterodimer. Importantly, orthosteric agonists and antagonists bind to GABA_B_R1, while PAMs bind to the GABA_B_R2 subunit. GABA_B_ receptors are primarily found in the cerebellum, prefrontal cortex, and thalamus, in addition to the interpeduncular nucleus and the olfactory nucleus (Bowery et al., 1987). Alcohol is known to interact with the GABA_B_ receptors in the brain, but the exact binding site and mechanism of action are not completely understood. GABA_B_ receptor-binding drugs have anti-convulsant and analgesic properties (Terunuma, 2018) and are also found to reduce craving and withdrawal symptoms in dependent individuals [for example, Baclofen (Logge et al., 2022)].

## 4. From alcohol use to alcohol use disorders – the GABAergic system

DSM-5 classifies substance-related disorders into substance-use disorders (SUD) and substance-induced disorders (intoxication, withdrawal, and other substance/medication-induced mental ailments). Clinically, SUDs occur in a range of severity based on a number of symptom criteria endorsed. Mild (2–3 symptoms), moderate (4–5), and severe (>5). The DSM-5 diagnostic criteria do not describe levels or types of alcohol use or alcohol use harms (American Psychological Association, 2013); however, for this review, we chose to include some of the most commonly used categories of this kind (e.g., binge alcohol use) for a better illustration of the AUD pathophysiology and the involvement of GABAergic system to align with clinical presentations of AUD and alcohol withdrawal. AUD encompasses various disorders characterized by different consumption patterns, impacting the brain and the GABAergic system. Alcohol consumption, including alcohol use not meeting the criteria for AUD, also impacts the GABAergic system. For example, minimal alcohol intake will enter the brain and target GABA_A_ receptors, causing a cascade of regulatory events, potentially leading to behavioral changes. When consumption becomes chronic, or during binge drinking episodes, the impact of alcohol on the brain is even more profound, triggering activation/inhibition of other biological pathways (as described earlier in Figure 1). Table 1 below summarizes alcohol use at different levels, explains the different considerations given for men and women, and highlights the impact on the GABAergic system and symptoms related to the use of alcohol.

**Table 1:** Alcohol consumption, symptoms, and the role of the GABAergic system.

Stage of alcohol use	Men	Women	Involvement of GABAergic system	Symptoms/Behavioral outcomes
Moderate use – low risk drinking	2 drinks/day or 28 g of ethanol/day*	1 drink/day or 14 g of ethanol/day*	Following acute ethanol ingestion, GABAA receptors are activated in basolateral amygdala and decreases glutamate action. Ethanol also downregulates extrasynaptic α4βδ–GABA_A_Rs in the hippocampus (Liang and Olsen, 2014).	Anxiolysis, sedation accompanied by decreased attention, alterations in memory, mood changes, and lethargy.
Use above low-risk drinking	**>**2 drinks/day and <14 drinks/week	>1 drink/day and <7 drinks/week	At low concentrations (10 mmol/L), GABAA receptors are activated in VTA, NAc, hypothalamus and hippocampus, while at concentrations higher than 13 mmol/L activates the mesolimbic reward pathway and increases the DA levels.	Increased voluntary ethanol ingestion
Binge drinking	>5 drinks/2 h Or > 60 g of ethanol/occasion	>4 drinks/2 h Or > 40 g of ethanol/occasion	Compensatory downregulation of cortical GABA levels and GABA_A_ receptors along with hyperexcitability (Marinkovic et al., 2022).	Insomnia, irritability, anxiety, autonomic hyperactivity and seizures
Heavy drinking	>5 drinks/day or > 15 drinks/week	>4 drinks/day or > 8 drinks/week	Heavy alcohol consumption causes increased internalization of α1 and α4 subunit-containing GABA_A_ receptors on hippocampal pyramidal cells thereby decreasing the availability of post-synaptic GABA_A_ receptors. Which in turn leads to increased alcohol consumption to attain the activation of desired GABA activity (Trudell et al., 2014).	Chronic downregulation of α1 and α4 subunit GABAA receptors may lead to increased alcohol tolerance, leading to dependence.
Dependence/Alcoholism	Compulsive drinking with increased alcohol tolerance		GABAergic hypofunction following chronic alcohol consumption leads to reduced GABAergic (via GABA_B_ receptors) inhibition of DA neurons in VTA leads to reward-associated alcoholism (Enoch, 2008).	Development of positive (pleasure) and negative (aversive – avoiding anxiety) reinforcement behaviors as the motivation to seek more alcohol.
Withdrawal (AWS)	Symptoms following the discontinuation or complete cessation of alcohol consumption		Chronic alcohol drinking increases GABA activity in comparison to glutamate (GABA > Glutamate). In the absence of alcohol (withdrawal), the GABA activity decreases but the increased glutamate (as compensation) levels remain about the same and leading Glutamate > GABA state.	Withdrawal causes hyperexcitability, elevated adrenergic system responses along with anxiety, insomnia and dysphoria.
Relapse	Spontaneous or delayed reoccurrence of alcohol drinking to avoid AWS or due to various internal or external stimuli		Hyperexcitability, seizures, and anxiety due to withdrawal-related GABA hypofunction can be the major reasons behind the relapse. Furthermore, reduced GABAergic and uninhibited DA transmission in NAc may lead to cue-induced/reward-based (craving) relapse (Heinz et al., 2009).	Relapse-related DA-reward “hijack” leads to the dysfunction of different domains of cognition.

A regular alcoholic drink = 10–14 g of ethanol. *on days when alcohol is consumed.

### 4.1. Occasional, moderate, and safe use of alcohol with low risk for AUD

The safe or moderate use of alcohol is considered with less than 2 drinks per day for men and 1 for women (0.02–0.04 g/dL blood alcohol concentration), where the risk for developing AUD remains low. Even with such use, the acute or low level of ethanol present in the system is enough to potentiate the action of GABA at GABA_A_ receptors, inducing relaxation. Even in rats, acute ethanol administration induces a state of anxiolysis driven by the potentiation of the GABA_A_ receptor in the basolateral amygdala, acting on multiple cell populations (Herman and Roberto, 2016). Low levels of ethanol already play a role in the expression and trafficking of GABA_A_ receptors in the brain by rapidly downregulating α_4_β_3_δ-GABA_A_ receptors in the hippocampus (Chandler et al., 2017). Expression of the α_1_β_3_γ_2_-GABA_A_ receptors is also downregulated after several hours of consumption, followed by an upregulation of α_4_β_3_γ_2_ and α_2_β_3_γ_1_ after a couple of days. This demonstrates the broad and long-lasting kinetics of an acute consumption of ethanol, which is reversible, but the recovery timeline is dose-dependent (Holford, 1987).

During medium-risk drinking, i.e., drinking episodes of alcohol when the volume of alcohol is consumed in a short period but not binge drinking (not more than 5 drinks in 2 h for men, 4 in 2 h for women, and < 0.08 g/dL) (World Health Organization, 2000), ethanol levels can range from 5 to 30 mmol/L. This potentiates the GABA_A_ receptors in the brain, decreasing excitatory glutamatergic neurotransmission and causing slight sedation, a feeling of relief, slight alteration of short-term memory, decreased attention, and potential mood changes (Liang and Olsen, 2014). Studies in rats have demonstrated that this dose level increases GABAergic firing rate and afferent-evoked synaptic response in the VTA, a central hub for dopaminergic projections in the brain, regulating motivation, cognition, reward valuation, and addiction. This impact on the VTA, potentially driven by changes in firing rates from the GABAergic system, contributes to increased alcohol intake (Tateno and Robinson, 2011).

Interestingly, preclinical studies using rats also demonstrated that reducing α_4_-subunit expression via a viral-mediated RNA interfering with the α_4_-protein synthesis in the NAc allowed for a reduction of self-administered ethanol. Similar results were observed when pharmacologically blocking the GABA_A_ receptors in the paraventricular nucleus of the hypothalamus, further confirming the role of GABAergic potentiation in increasing alcohol intake and seeking behaviors (Li et al., 2011).

### 4.2. At-risk drinking patterns

Greater than the threshold set for safe and moderate use described above, consumption of alcohol is considered at risk (NIAAA, 2018). In this case, ethanol induces GABA_A_ receptor activation in the VTA, NAc, hypothalamus, and hippocampus, causing an overall imbalance in excitation/inhibition, leaning toward increased inhibition. At a certain point, thought to be above 13 mmol/L (Liang and Olsen, 2014), the reward pathways of the mesolimbic system are directly and indirectly activated (as described in the previous section on *Impact of Ethanol on the Brain*), allowing dopamine release, which fosters the development of addictive properties of alcohol consumption.

### 4.3. Alcohol use disorder

AUD is considered when the drinking pattern is above established standards, either due to volume or frequency of intake. One tool used worldwide to identify AUD is the Alcohol Use Disorder Identification Tool (AUDIT), developed by WHO (Higgins-Biddle and Babor, 2018). While the classification of AUD has changed over the years and is country-dependent, most medical and addiction professionals frequently break AUD into two categories: binge and heavy drinking (Kranzler and Soyka, 2018).

Binge drinking is the acute consumption of large amounts of alcohol (for example, five or more drinks in less than 2 h for men and four or more for women, leading to >0.08 g/dL of blood alcohol concentration). Binge drinking leads to cognitive deficits, reduced inhibition, and reduced ability to control alcohol intake voluntarily, thereby increasing the chances of developing more frequent AUD in the future (Chmielewski et al., 2020). Risk factors for binge drinking include age, male sex, alcohol consumption at a young age, a patient’s state of mental health, and genetic susceptibility (NIAAA, 2020).

Preclinical studies of psychological changes and alcohol consumption have determined that in young rats (postnatal days 28–42), binge drinking induces a state of anxiety-like behavior and leads to alcohol dependence in adulthood (Pandey et al., 2015). Stress and withdrawal-induced anxiety are correlated to increased voluntary ethanol drinking in alcohol-preferring rats (Meyer et al., 2013), and chronic psychosocial stressed male mice showed increased voluntary ethanol drinking (Bahi, 2013). Human magnetic resonance spectroscopy studies have shown that cortical GABA levels are reduced in young adult binge drinkers (Marinkovic et al., 2022). Following acute high-dose ethanol administration in rats, thalamic α_4_-GABA_A_ receptor levels were regulated temporally, as a decrease was observed at 2 h followed by a delayed transient increase (Werner et al., 2016). Other studies using a transgenic dopaminergic D3 receptor knockout mouse model combined with an α6-GABA_A_ receptor ligand (RO 15–4,513) also showed that increased GABAergic inhibition in the NAc contributes to reducing binge drinking, confirming the critical role of GABAergic neurotransmission in reducing alcohol intake (Leggio et al., 2019).

Heavy drinking is defined as drinking more than recommended during a week, leading to 0.1–0.2 g/dL of blood alcohol concentration, depending on the number of drinks. For a man, having more than 15 standard alcoholic drinks weekly is considered heavy drinking. For women, having more than 8 drinks a week meets the criteria for heavy drinking (NIAAA, 2018). Heavy drinking leads to increased neuronal atrophy and reduces white matter fiber integrity (Daviet et al., 2022), associated with increased risk for dependence, anxiety, depression, cognitive deficits, altered control over drinking habits, cardiovascular diseases, and other health risks.

Studies have shown that the behavioral changes are primarily due to the plastic changes of GABA_A_ receptors that occur after chronic ethanol exposure, which include significantly reduced post-synaptic α_1_ and increased α_4_-containing GABA_A_ receptors. The subunit composition of GABA_A_ receptor subtypes is expected to determine their physiological properties and pharmacological profiles. An in-depth study of GABA_A_-subunits using genetically engineered mice has shown that the α_1_ subunit involves sedation, anti-convulsant activity, anterograde amnesia functions, etc., while the α_4_ subunit is involved in changes in mood and anxiety. Thus, these GABA_A_ receptor subunit composition changes are a mechanism underlying the behavioral changes after chronic ethanol exposure, which leads to additional risks of developing dependence. Heavy drinking triggered by chronic stress and any induced anxiety is an additional risk factor for developing alcohol dependence, observed in animal models and humans (McCaul et al., 2017). Conversely, stopping or reducing alcohol consumption, in turn, aggravates stress or anxiety due to an overall imbalance in brain homeostasis (Schmidt et al., 2016).

### 4.4. Chronic/daily alcohol use leading to dependence

With chronic alcohol consumption comes an increased risk for reward-associated habitual alcohol abuse, pronounced craving behavior for alcohol, and inability to stop seeking alcohol. This is usually highly linked to the development of dependence, a severe form of AUD that occurs when a person develops tolerance to the effect of alcohol and, therefore, seeks further alcohol consumption to prevent experiencing withdrawal symptoms. Alcohol dependence is a serious condition that requires comprehensive treatment to address the physical, emotional, and behavioral aspects of AUD.

Postmortem studies found a loss of GABAergic markers in the human brains of adults with alcohol dependence, particularly in men (Behar et al., 1999; Dodd et al., 2006). Transcranial magnetic stimulation (TMS) studies also demonstrated that chronic alcohol dependence has some level of impact on GABA_A_ and GABA_B_ receptor function, which seems to vary from study to study (Mohammadi et al., 2006; Ziemann et al., 2015). Several studies found no effects on short-interval cortical inhibition or TMS-evoked N45 potential (Conte et al., 2008; Nardone et al., 2010; Mon et al., 2012; Naim-Feil et al., 2016), thought to index GABA_A_ receptor function. However, other studies found a general decrease in GABA levels (Prisciandaro et al., 2019; Shyu et al., 2022), including in youth with alcohol dependence (Kaarre et al., 2018). Given the dynamic nature of alcohol’s effects on GABA, the GABA levels depend on several states (e.g., recently detoxified or more prolonged abstinence) and traits (e.g., age). One report on long-interval cortical inhibition thought to index GABA_B_ showed decreases in alcohol-dependent patients (Naim-Feil et al., 2016).

Multiple preclinical studies demonstrated that chronic ethanol consumption alters GABA_A_ receptor plasticity, leading to ethanol dependence (Olsen and Liang, 2017). Other preclinical studies established that general GABA_A_ receptor expression and function changes in cases of alcohol dependence, both synaptically and extra-synaptically, in brain regions highly involved in establishing dependence and symptom emergence (i.e., the cortex, hippocampus, and central amygdala). This translates into a general loss of phasic and tonic GABAergic inhibition, tolerance to ethanol, and cross-tolerance to benzodiazepines and other sedative-hypnotics acting on GABA receptors (Kumar et al., 2009; Olsen and Liang, 2017; Bohnsack et al., 2018).

With such alteration in overall GABAergic functioning, a drastic imbalance in excitation/inhibition develops across multiple brain regions [medial prefrontal cortex (Pleil et al., 2015)], amygdala circuit (Herman et al., 2016; Herman and Roberto, 2016; Hughes et al., 2019), intrahippocampal circuits (Liang et al., 2004), and VTA circuits (Arora et al., 2013) causing a decrease in inhibitory control in multiple neurotransmitter firing activity, leading to the emergence of various behavioral changes including cognitive deficits, seeking behavior, humor changes, and others (Morrow et al., 2020).

Chronic alcohol consumption in heavy drinking, dependence, and associated GABA_A_ plasticity changes also lead to DA release changes in the reward neurocircuitry. During acute alcohol withdrawal, changes occur, such as upregulation of α_4_-containing GABA_A_ receptors and downregulation of α_1_- and α_3_-containing GABA_A_ receptors (Liang and Olsen, 2014). GABA_A_ receptor downregulation may contribute to anxiety and seizures of withdrawal. During withdrawal periods, rats show a significant decrease in DA and serotonin levels in the reward neurocircuitry commonly associated with dysphoria, depression, and anxiety disorders. These psychological changes may also contribute to ethanol-seeking behavior, again demonstrating the complexity of changes induced by chronic alcohol consumption.

## 5. Existing interventions

Existing therapeutic interventions for AUD and alcohol withdrawal have attempted to harness the various CNS systems on which alcohol acts to limit the harms associated with alcohol consumption. The existing therapeutic interventions have diverse efficacy levels, various side effects, and contraindications (Table 2). Several clinical trials have shown the efficacy of certain pharmacotherapies that are approved by regulatory agencies for treating AUD or withdrawal and that are used off-label (Carpenter et al., 2018; Sloan et al., 2020).

**Table 2:** Existing pharmacological therapeutics to treat AUDs, their efficacy, and limitations.

Drug name	Indications	Mechanism of action (MoA)	Effects on AUDs	Adverse drug reactions/side effects	Contraindications	References
Acamprosate	AUD in Europe and North America (FDA approved)	Unknown. ↓ Glutamate during alcohol withdrawal via NMDA modulation. Potentiates GABA_A_ receptors through GABA_B_ receptor inhibition.	Decreases alcohol craving and prevents relapse. Reduce alcohol consumption and increases abstinence when combined with psychosocial support.	Suicidality, amnesia, anxiety, depression, somnolence, nausea, vomiting, abdominal pain, pruritis, and rashes.	Severe renal diseases and dose lowered in mild renal diseases.	Franck and Jayaram-Lindström (2013), Sloan et al. (2020)
Baclofen	AUD in France (Off label in other countries) Primary indication: Centrally acting muscle relaxant.	GABA_B_ agonist ↑ K^+^ and ↓Ca^2+^ influx in neurons. ↓ dopamine release.	Can reduces craving and withdrawal symptoms in dependent individuals. (Inconsistent findings).	Drowsiness, sedation, dizziness, headache, confusion, muscle stiffness, excessive perspiration, itching, abnormal muscle movements, numbness, and slurred speech.	Cardiac disease Respiratory disease Severe psychiatric disorders Liver or kidney disease (require adjusted dosing)	Garbutt (2019), Mason (2017), Rolland et al. (2020), Sloan et al. (2020)
Disulfiram	AUD (second-line treatment)	Irreversible inhibitor of acetaldehyde dehydrogenase.	Elevated levels of acetaldehyde lead to severe adverse reactions, limiting patient to consume further alcoholic beverages.	Optic neuritis, psychosis, hepatotoxicity, peripheral neuropathy Metallic aftertaste, dermatitis, moderate to severe drowsiness, hepatitis, neuropathy, headaches, and confusion.	Use of metronidazole, paraldehyde or alcohol-containing products Cardiac Disease Hepatic Disease Diabetes Pregnancy.	Kranzler and Soyka (2018), Sloan et al. (2020)
Nalmefene	Controlled drinking (Australia and Europe)	Antagonist of μ and δ opioid receptors Partial agonist at the κ receptor. ↓ Dopamine release in the nucleus accumbens.	It modulates dopaminergic NAc circuitry via kappa receptor activation to reduce dependence by decreasing the rewarding and craving effects of alcohol. It can help control alcohol consumption with psychosocial support.	Nausea, dizziness, insomnia, headache, vomiting, fatigue, and drowsiness.	It may elicit opioid withdrawal in patients taking opioids or recently suffering from opioid addiction.	López-Pelayo et al. (2020)
Naltrexone	AUD (first-line treatment and FDA-approved)	Competitive antagonist at the μ opioid receptor with mild antagonistic activity at the δ and κ opioid receptors.	Reduce cravings and feelings of euphoria associated with AUD. Reduces the chances of relapsing. Reduces opioidergic-dependent dopamine activity in the mesolimbic system to reduce the rewarding effects.	Hepatotoxicity, precipited withdrawal, depression and suicidality Somnolence, nausea, vomiting, anorexia, insomnia, headache, dizziness, gastrointestinal discomfort, including abdominal cramps and diarrhea.	Concurrent opioid use. Liver failure or liver disease Bleeding or coagulation disorder	Liang and Olsen (2014), Sloan et al. (2020)
Gabapentin	AUD (Off label) Primary indication: neuropathic pain, neuralgia, and seizure	GABA analog but unknown MOA. supposed main target: α2δ1 voltage-gated Ca^2+^ channel ↑ GABA concentrations in the brain.	Reduces cravings, decreases the risk of relapse to heavy drinking, and increases abstinence. More significant effects are seen when taken in combination with Naltrexone.	Anaphylaxis, suicidality Dizziness, somnolence, ataxia, dry mouth, weight gain, fatigue, nystagmus, and tremor.	Severe renal disease	Anton et al. (2020), Cai et al. (2012), Sloan et al. (2020)
Topiramate	AUD (Off label) Primary indication: Anti-convulsant	↑ GABA_A_ receptor activity. ↓ Glutamate release via AMPA receptor. ↓ Dopamine release in the nucleus accumbens.	Reduces craving, reward, and the risk of relapse. Decreases withdrawal symptoms by mediating hyperexcitability in the brain, thereby increasing abstinence.	Nephrolithiasis, Hyperammonemia, suicidality, hyperthermia, metabolic acidosis, glaucoma Cognitive dysfunctions, Paresthesia, dysgeusia, anorexia, anorexia, weight loss, nervousness, dizziness, and somnolence.	Pregnancy	Burnette et al. (2022), Paparrigopoulos et al. (2011), Sloan et al. (2020), Wenzel et al. (2006)
Benzodiazepines	Withdrawal	Allosteric modulator of GABA_A_ receptors (α1/2/3/5β1/3γ2)	Treats acute alcohol withdrawal symptoms. Prevents withdrawal-induced seizures.	Sedation, drowsiness, ataxia, and anterograde amnesia.	Current consumption of alcohol Renal or liver disease	Nelson et al. (2019)

### 5.1. Non-GABAergic pharmacologic interventions

Disulfiram has been an FDA-approved drug used to treat AUD since 1951. It inhibits the acetaldehyde dehydrogenase enzyme involved in ethanol metabolism, leading to higher plasma concentrations of acetaldehyde, which induces unpleasant side effects if a patient consumes alcohol while taking this medication, preventing further drinking. Disulfiram-induced reactions can include hepatotoxicity and death, which is why disulfiram needs to be used with caution (Kranzler and Soyka, 2018; Stokes and Abdijadid, 2022). Nowadays, the most used pharmacotherapy is naltrexone (commercialized under the brand name Revia®), a competitive μ opioid receptor antagonist and a partial antagonist of the δ and κ opioid receptors (Liang and Olsen, 2014; Sloan et al., 2020; Singh and Saadabadi, 2023). It decreases craving by reducing the rewarding and euphoric effects of alcohol and is one of the few AUD pharmacotherapies approved by the FDA. It is generally well tolerated but has minor side effects (Singh and Saadabadi, 2023).

Acamprosate is an FDA-approved drug used in Europe and North America for alcohol craving and relapse prevention (Franck and Jayaram-Lindström, 2013; Kalk and Lingford-Hughes, 2014). Although its exact mechanisms are unknown, it decreases glutamate during alcohol withdrawal through NMDA receptor modulation and indirectly potentiates GABA_A_ receptors. Acamprosate is generally well tolerated (Kalk and Lingford-Hughes, 2014).

Nalmefene is another antagonist of the μ and δ opioid receptors but is a partial agonist at the κ receptor. It is currently approved for AUD indication in Australia and Europe. Nalmefene decreases dopamine release in the NAc and reduces alcohol dependence and consumption by decreasing the rewarding and craving effects of alcohol (Paille and Martini, 2014). It can help control alcohol intake and has shown better results in those benefiting from psychosocial support. It has mild side effects, which generally disappear with time (Paille and Martini, 2014; López-Pelayo et al., 2020).

### 5.2. GABAergic pharmacologic interventions

Baclofen is only approved for the treatment of alcohol withdrawal in France (Garbutt, 2019). Despite multiple trials supporting its efficacy in reducing the risk of relapse and increasing abstinence days (Agabio et al., 2023), its efficacy remains controversial, and systematic reviews consider the evidence of its efficacy insufficient (Jonas et al., 2014). It acts as an agonist at the GABA_B_ receptor and decreases dopamine release in the mesolimbic system, which reduces craving and withdrawal symptoms in dependent individuals. Baclofen has multiple side effects, limiting its use (Romito et al., 2021).

Gabapentin is a GABA analog used as an anti-epileptic medication for over 30 years. Clinical trials have shown dose-dependent efficacy in reducing craving, reducing anxiety, and facilitating abstinence (Anton et al., 2020). However, some studies also raise concerns due to its sedating properties and documentation of extra-medical use of this medication (Modesto-Lowe et al., 2019; Weresch et al., 2021). It was also found that Gabapentin causes respiratory depression when used alone and increases the risk of opioid-related deaths when combined with opioids (Gomes et al., 2017). Despite being a GABA analog, its mechanism of action is still unclear and seems unrelated to GABAergic modulation. Its main target seems to be the α_2_δ-subunit of the voltage-gated calcium channel. It also increases GABA concentrations in the brain (Cai et al., 2012).

Topiramate is not yet approved by the FDA for the treatment of AUD. Still, clinical trials have demonstrated reductions in craving and risk of relapse and increasing abstinence (Kranzler et al., 2014; Manhapra et al., 2019; Wetherill et al., 2021). It is an approved anti-convulsant for treating epilepsy and seems to act through GABA_A_ receptor modulation (Fariba and Saadabadi, 2022). It also binds the AMPA receptor to decrease glutamate release and decreases dopamine release in the NAc. It has some side effects, including paresthesia, dysgeusia, anorexia, and cognitive impacts such as slowing mental and physical activity and trouble concentrating or attention (Wenzel et al., 2006).

Benzodiazepines (BZ) are allosteric modulators of the GABA_A_ receptor that bind to the α_1, 2, 3, 5,_ and γ subunits. They enhance the activity of GABA when binding at its receptor and are recommended in managing acute alcohol withdrawal (Nelson et al., 2019), but not for the treatment of AUD itself. They can lead to sedation, ataxia, anterograde amnesia, and have abuse potential (Engin, 2022). Alcohol delays the metabolism of BZ (Hoyumpa, 1984), prolonging its bioavailability, causing psychomotor impairment, and increasing the risk of overdosing of BZ. Studies showed that BZ also modulates part of ethanol’s reinforcing and/or aversive properties. BZ and ethanol co-consumption is also known to amplify the effect of alcohol.

### 5.3. Psychotherapeutic interventions

In contrast to pharmacological interventions, Cognitive Behavioral Therapy (CBT) is a form of psychotherapy that involves challenging automatic thoughts, cognitive distortions, existing beliefs, and problematic behaviors (Chand et al., 2023). It is one of the most studied forms of treatment for SUD and has the most support from evidence-based studies. Adults with problematic drinking who received CBT showed decreased alcohol consumption, and newer variants of CBT, such as virtual reality-assisted CBT (Thaysen-Petersen et al., 2023), appear to be more successful than traditional methods (Carroll and Kiluk, 2017).

Motivational Enhancement Therapy (MET) is another psychosocial treatment that applies principles from motivational psychology. MET is often the foundation of brief interventions for risky alcohol use, and indeed, protocols can be very short, requiring only a few sessions of client-centered interventions (Ceci et al., 2022). MET focuses on identifying a reason for a change in alcohol consumption, but outcomes vary substantially with commitment and readiness to change to have an impact (Hodgins et al., 2009).

However, existing therapeutic options have shown limitations. Some drugs, repurposed from other indications, show direct or indirect activity in the GABAergic system (Gabapentin, topiramate, and baclofen). The GABAergic system is a key player in the pathophysiology of AUD and alcohol withdrawal and is a desirable target for drug development (Liang and Olsen, 2014; Mirijello et al., 2015). Indeed, the previous sections showed how intertwined central pathways are in the context of ethanol consumption and how instrumental the GABAergic system is in modulating most of the effects, directly or indirectly. However, AUD is broad and can vary in expression in multiple ways (volume consumed, acute or chronic consumption, etc.). Therefore, the impact of ethanol on the GABAergic system may vary depending on the manifestation of AUD, and different interventions acting on different aspects of the GABAergic system may be required to elicit optimal outcomes in treating AUD or alcohol withdrawal. The following sections will present novel GABAergic interventions currently being investigated.

## 6. GABAergic interventions in preclinical models and their impact on alcohol-related symptoms: reconciling risk and benefits

### 6.1. GABA_A_: involvement in AUD and therapeutic potential

Since ethanol facilitates the activity of GABA and has such a large effect on GABAergic receptor expression and function, it can be difficult to anticipate what impact a GABAergic drug would have on individuals with AUDs. Benzodiazepines (BZ), binding at the interface between α_1-2-3-5_ and γ subunits of the GABA_A_ receptors, are known enhancers of phasic GABAergic inhibition across brain regions and induce internalization of synaptic GABA_A_ receptors (Gallager et al., 1984; Tehrani and Barnes, 1997). Therefore, BZs promote the mechanisms leading to some ethanol-induced deficits in GABAergic inhibition. However, BZs have beneficial effects in the context of acute withdrawal symptoms as they act as a substitute for ethanol and can help individuals in withdrawal re-establish a new excitation/inhibition balance without alcohol (refer to Figure 1B).

In recent years, BZ-derivatives acting preferentially at selected α-subunits were developed and tested in preclinical models for their activity on ethanol self-administration and craving behaviors (Table 3). Activation of α_2_/α_3_-GABA_A_ receptors by the HZ-166, XHe-II-053, YT-III-31, or YT-III-271 PAMs in ethanol discrimination studies augmented the reinforcing effects of ethanol via increasing the self-administration in rhesus monkeys (Berro et al., 2019). These findings are aligned with clinical evidence that demonstrated a positive association of both the GABRA2 and GABRA3 gene expression with an increased risk for developing alcoholism (Covault et al., 2004; Enoch, 2008; Soyka et al., 2008; Mallard et al., 2018). Similarly, potentiation of the α_5_-GABA_A_ receptor via QH-ii-066 administration was also shown to enhance the reinforcing effects of alcohol in non-human primates, while using an inverse agonist at the α_5_-GABA_A_ receptor (Xli-093) inhibited such reinforcement effects (Rüedi-Bettschen et al., 2013). Consistently, intra-hippocampal infusions of an α_5_-GABA_A_ receptor inverse agonist RY023 reduced ethanol-maintained responses in a dose-dependent manner, suggesting that the α_5_-GABA_A_ receptors in the hippocampus play an important role in regulating ethanol-seeking behaviors (June et al., 2001). This was further supported by studies using the partial α_5_-GABA_A_ receptor inverse agonist Ro 15–4,513, by the selective α_5−_GABA_A_ inverse agonist (α5IA-II) (Stephens et al., 2005) and by the use of the α_5_-GABA_A_ receptor knockout mice model showing reduced ethanol preference (Boehm II et al., 2004; Stephens et al., 2005).

**Table 3:** Role of GABA_A_ receptor subunits in alcohol abuse-related effects (Berro et al., 2019).

Drug candidate	MoA	Outcomes of the treatment on AUDs symptoms	Reference
HZ-166	α2 and α3 GABA_A_ PAM	Following the administration, the drug increased the alcohol-related lever pressing and significantly increased the ethanol self-administration (reinforcement). The effects are similar to the ethanol indicating the respective subunit’s involvement in the dependence behavior responsible for prolonged ethanol intake.	Berro et al. (2019), Rüedi-Bettschen et al. (2013)
YT-III-31 & YT-III-271	Selective α3 GABA_A_ PAM		
QH-ii-066	α5-GABA_A_ receptor-preferring PAM		
L-838417	α1-sparing, functionally selective partial PAM of α2/3/5-GABA_A_ receptors		
YT-III-271	Selective α3 GABA_A_ PAM		
XHe-II-053	Selective α2 and α3 GABA_A_ PAM		
XLi-093	α5 antagonist	Decreased ethanol discrimination and reinforcements.	Rüedi-Bettschen et al. (2013)
RY023	α5 inverse agonist	Intrahippocampal administration decreases ethanol-maintained responses in lever pressing task	June et al. (2001)
Ro 15–4,513	BZ reverse agonist and α_4,6_-agonist	Reduction of operant response for ethanol	Stephens et al. (2005)
α5IA-II	α5 inverse agonist	Decreased lever pressing in rats with alcohol dependence	Stephens et al. (2005)

However, the studies mentioned above all evaluated the impact of positive modulation of the α_x-_GABA_A_ receptor in the context of alcohol consumption or alcohol discrimination when the system is already sensitized to further GABAergic activity (Figure 1B – central panel). However, it remains unclear how such modulation would play in the context of withdrawal when the system is deficient in GABAergic regulation, which is, in turn, causing craving behaviors. Knowing the anti-craving effect of BZ (Nelson et al., 2019), one could expect that the α_2_-, α_3_- or α_5_-PAMs can contribute to the anti-craving effect of BZ in a brain system during a withdrawal state and could further elicit beneficial effects without the side effects observed with benzodiazepines.

While BZ and derivatives bind and act at the interface between α_1-2-3-5_ and γ subunits, neurosteroids bind between α and β subunits of the GABA_A_ receptors. Furthermore, such binding is greatly facilitated by the presence of the δ subunit in the pentamer (Gatta et al., 2022; Figure 2B). Neurosteroids are potent and effective neuromodulators synthesized from cholesterol in glial and neuronal cells of the central (CNS) and peripheral nervous systems (PNS). They act at extrasynaptic receptors, facilitating tonic inhibition (Chen et al., 2019; Belelli et al., 2022). With acute alcohol intake, the cerebral levels of allopregnanolone were found to be increased, whereas its levels were reduced during chronic alcohol consumption and withdrawal (Romeo et al., 1996). In addition, stimulation of neurosteroidogenesis by metyrapone was found to reduce cocaine intake in rats (Goeders and Guerin, 2008), and one could suppose a similar effect for alcohol intake.

Recent studies found that allopregnanolone has antidepressant properties for women with postpartum depression (Pinna et al., 2022), a disorder with reduced GABAergic function (Prevot and Sibille, 2021). Therefore, with their action of the GABAergic system, and their involvement in arousal, cognition, emotion, and motivation, neurosteroids may hold therapeutic potential in treating AUD (Zorumski et al., 2013; Gatta et al., 2022), and such effects are being investigated (Morrow et al., 2020; Mounier et al., 2021).

### 6.2. GABA_B_: involvement in AUD and therapeutic potential

The involvement of GABA_B_ receptors in the development of AUD is still unclear. However, studies in clinical populations (using Baclofen) and animals [experimental candidates listed in Table 4 (Maccioni and Colombo, 2019)] showed that GABA_B_ receptor modulation was beneficial in AUD management. For instance, rats receiving baclofen showed reduced hyper-locomotion caused by acute alcohol administration (Besheer et al., 2004), and reduced anxiety-like behavior and tremors following chronic alcohol withdrawal (Knapp et al., 2007). Table 4 includes a list of GABA_B_ PAMs such as CGP7930, GS39783, BHF177, Rac-BHFF, ADX71441, CMPPE, COR659, and ORM-27669 that were primarily studied in rodent models and were found to be beneficial in AUDs.

**Table 4:** GABA_B_-positive allosteric modulators under development for AUD.

Drug candidate	Outcomes of the treatment	Reference
GS39783	Attenuates (repeated dosing) and reduces (acute treatment) ethanol-induced hyper locomotion at (30 mg/kg; ip)	Kruse et al. (2012)
	Dose-dependently suppressed the acquisition of alcohol-drinking behavior. Also, reduced daily alcohol intake by 30–40%.	Orrù et al. (2005)
	Reduced alcohol intake in a dose-dependent manner.	Colombo et al. (2015)
	Reduced binge-like alcohol drinking	Linsenbardt and Boehm Ii (2014)
	Decreases self-administration of alcohol	Lorrai et al. (2019)
		Maccioni et al. (2017)
Rac-BHFF	Repeated dosing reduced alcohol-reinforcing properties. Also, prevented tolerance development.	Maccioni et al. (2015)
	7 consecutive dose administrations reduced daily alcohol intake in Sardinian alcohol-preferring rats.	Loi et al. (2013)
	At non-sedative doses, it reversed ethanol-induced plasticity and reduced ethanol drinking.	de Miguel et al. (2019)
ORM-27669	Pretreatment with ORM-27669 only reversed ethanol-induced neuroplasticity and attenuated ethanol drinking	de Miguel et al. (2019)
CGP7930	Dose-dependently suppressed the acquisition of alcohol-drinking behavior. Also, reduced daily alcohol intake by 30–40%.	Orrù et al. (2005), Maccioni et al. (2018)
ADX71441	Dose-dependent reduction of alcohol self-administration and suppressed stress-induced relapse.	Augier et al. (2017)
	Reduced alcohol drinking in intermittent and drink-in-the-dark (DID) models.	Hwa et al. (2014)
CMPPE	Dose-dependent reduction in self-administration and cue-induced reinstatement of alcohol seeking in alcohol-preferring rats.	MacCioni et al. (2009)
BHF177	Dose-dependently reduced alcohol self-administration.	MacCioni et al. (2009)
	Acute administration at non-sedative doses, it selectively reduced alcohol intoxication in binge-like drinking experiments.	Lorrai et al. (2022)
ASP8062	Reduced the alcohol self-administration but did not alter alcohol-related locomotion.	Haile et al. (2021)
KK-92A	Dose-related suppression in alcohol self-administration	Maccioni et al. (2022)

## 7. Novel therapeutic agents targeting the GABAergic system in clinical trials

### 7.1. Pharmacological interventions

With the increased characterization of the impact of alcohol on the GABAergic system and the increasing characterization of the link between GABAergic functions, receptor subtype, and symptom relief in the context of AUD, more clinical trials are being initiated to investigate how GABAergic modulation can contribute to better treatment of AUDs and alcohol withdrawal (Table 5). Interventions acting on GABA_A_ receptors are investigated in multiple clinical trials. For example, DZ is already the standard of care for reducing withdrawal symptoms. Midazolam, another benzodiazepine, and propofol, a GABA_A_ receptor agonist, were withdrawn from Phase 4 studies in 2016 due to logistical reasons. They were studied for their potential effect on stress response and immune functions in mechanically ventilated patients with AUDs.

**Table 5:** GABA modulators in clinical trials for AUD and alcohol withdrawal treatment (Source: clinicaltrials.org).

Drug candidate and details of clinical trial	MoA	Indications	Current status	Reference
**ASP8062** Sponsors: NIAAA & Astellas Pharma Inc. Trial No. NCT05096117	GABA_B_-positive allosteric modulation	AUD, alcohol craving	Phase 2	Walzer et al. (2020)
**Brexanolone** Sponsors: Yale University, NIAAA & Sage Therapeutics Trial No. NCT05223829	GABA_A_ targeting neurosteroid	Stress-induced alcohol use in men and women with PTSD	Phase 1	Patatanian and Nguyen (2022)
**Propofol** **Midazolam** Sponsor: Virginia Commonwealth University Trial No. NCT00871039	GABA_A_-positive allosteric modulation GABA_A_-positive allosteric modulation	Stress response and immune function in mechanically ventilated patients with AUD	Withdrawn due to logistical purposes in 2016	Marik (2004), Zaporowska-Stachowiak et al. (2019)
**Baclofen** Sponsor: Universitair Ziekenhuis Brussel Trial No. NCT03293017	GABA_B_ agonist	Management of acute alcohol withdrawal – comparison with Diazepam	Phase 4	De Beaurepaire (2018), Dhaliwal et al. (2022)
**Valproate** **Lorazepam** Sponsor: CAMC Health System Trial No. NCT03235531	Inhibitor of GABA metabolism and GABA reuptake (so increases GABA levels) GABA_A_-positive allosteric modulation	Ethanol withdrawal syndrome, comparison with BZ (Lorazepam)	Phase 4	Ghiasi et al. (2023), Johannessen and Johannessen (2003)
**Disulfiram + Lorazepam** Sponsor: University of New Mexico and NIAAA Trial No. NCT00721526	Irreversible inhibitor of acetaldehyde dehydrogenase. + GABA_A_-positive allosteric modulation	Combination therapy for patients with alcohol dependence and anxiety disorder	Open label Phase 4 Completed	Bogenschutz et al. (2016)

Brexanolone, a GABA_A_-targeting neurosteroid, is about to start recruiting for a Phase 1 study to demonstrate safety before assessing efficacy in participants with AUD and PTSD. Brexanolone is already approved for treating postpartum depression (Morrow et al., 2020).

Baclofen, a GABA_B_ agonist, is currently under Phase 4 to assess its efficacy in managing acute alcohol withdrawal. As mentioned in Table 1, baclofen is already approved in France for reducing craving and withdrawal syndrome, but some literature suggests its efficacy for this indication is limited (Cooney et al., 2019). ASP8062, a GABA_B_-PAM, is currently investigating the efficacy of 2 weeks of treatment in a Phase 2 study in participants with moderate AUD at reducing alcohol cravings. Preclinical studies in rats showed promising effects in reducing alcohol self-administration without side effects observed with baclofen (Haile et al., 2021), and phase 1 studies in humans confirmed the safety of ASP8062 (Ito et al., 2022).

The antiepileptic valproate also acts indirectly on the GABAergic system by blocking the metabolism of GABA and by blocking GABA reuptake, increasing GABA levels in the brain (Janmohamed et al., 2020). Clinical trials are ongoing to determine the efficacy of valproate treatment at reducing ethanol withdrawal, compared to benzodiazepines, here lorazepam. Lorazepam was also used in another open-label clinical trial completed in 2013 to assess the efficacy of a combination with disulfiram. Reports showed a significant reduction in anxiety, depression, and craving; such effects were observed 24 weeks after intervention (Bogenschutz et al., 2016).

### 7.2. Non-pharmacological interventions – rTMS

Repetitive transcranial magnetic stimulation, i.e., rTMS, is a noninvasive neurostimulation modality delivering focused magnetic field pulses to the cortex that modulate cortical activity. Treatment sessions are generally delivered daily over several weeks, which results in the induction of long-term changes in cortical excitability through neuroplasticity. This includes modulation of the implicated neurocircuits underlying alcohol use disorder and is under investigation as a potential treatment. Enduring changes in cortical activity (namely inhibition and excitation) resulting from rTMS have implications for enduring changes in GABA activity (Daskalakis et al., 2006). Over a decade ago, the first published clinical trial demonstrated efficacy in reducing cravings in adults with AUD over a sham-control condition (Mishra et al., 2010). Since then, the majority of trials have delivered rTMS over the left or right dorsolateral prefrontal cortex, with a recent meta-analysis showing a signal for reduced alcohol craving with rTMS treatment (Sorkhou et al., 2022), potentially driven by the impact of rTMS on GABAergic signaling. However, most RCTs have been small single-center trials, and given the substantial heterogeneity in parameters utilized across studies, the optimal protocol has not yet been determined.

Additionally, there is growing interest in using deep rTMS™ using coils (H Coils) that can induce a broader electrical field within the cortex. For example, a recent RCT using rTMS with an H7 Coil stimulating the bilateral medial prefrontal cortex and anterior cingulate cortex showed positive results in reducing craving and alcohol consumption in treatment-seeking patients with AUD (Harel et al., 2022). Moreover, another trial that utilized a coil that stimulates the bilateral lateral PFC and insula showed efficacy for nicotine dependence in a large, definitive, multi-site RCT that subsequently paved the way for FDA clearance for this indication (Zangen et al., 2021), demonstrating the first time FDA cleared indication for any substance use disorder. Taken together, further exploration of the therapeutic potential of rTMS for AUD is warranted. Given the well-described link between GABA dysfunction in AUD and rTMS effects on the GABAergic system, it will be important to explore whether biomarkers of GABAergic functions can serve as mediators or moderators of rTMS efficacy.

## 8. Conclusion

Alcohol use-related disorders are significant risk factors for other high mortality-causing diseases. Although the mechanisms are elusive, the GABAergic system’s involvement seems critical in AUD development. Currently, GABAergic drugs are used in the second or third line of treatment of AUD and mitigation of alcohol withdrawal. Studies indicate that pharmacological modulation of GABA receptors may be a promising therapeutic option in achieving long-term abstinence by decreasing the daily alcohol intake and withdrawal effects. However, extensive research is needed in this line to uncover the pharmacological potential of the GABAergic system in managing alcohol use-related disorders.

## Author contributions

RD and TP conceptualized the review. RD, CP-L, and TP wrote the review, collected sections, and contributions from other co-authors, and prepared the figures and tables. VT, MS, YN, PP, AR, KS, DV, DB, IB, NB, PG, EV, OM, ES, and LQ provided content and critical revision of review drafts. All authors approved the final version of the manuscript.

## References

[ref1] Abrahao K. P. Salinas A. G. Lovinger D. M. (2017). Alcohol and the brain: neuronal molecular targets, synapses, and circuits. Neuron 96, 1223–1238. doi: 10.1016/j.neuron.2017.10.032, PMID: 29268093 PMC6566861

[ref2] Addolorato G. Leggio L. Hopf F. W. Diana M. Bonci A. (2012). Novel therapeutic strategies for alcohol and drug addiction: focus on GABA, ion channels and transcranial magnetic stimulation. Neuropsychopharmacology 37, 163–177. doi: 10.1038/npp.2011.216, PMID: 22030714 PMC3238087

[ref3] Agabio R. Saulle R. Rösner S. Minozzi S. (2023). Baclofen for alcohol use disorder. Cochrane Database Syst. Rev. 2023:CD012557. doi: 10.1002/14651858.CD012557.pub3, PMID: 36637087 PMC9837849

[ref4] American Psychological Association. (2013). Diagnostic and statistical manual of mental disorders: DSM-5™, 5th Edn Arlington, VA: American Psychiatric Publishing, Inc., 947.

[ref5] Anton R. F. Latham P. Voronin K. Book S. Hoffman M. Prisciandaro J.. (2020). Efficacy of gabapentin for the treatment of alcohol use disorder in patients with alcohol withdrawal symptoms: A randomized clinical trial. JAMA Intern. Med. 180, 728–736. doi: 10.1001/jamainternmed.2020.0249, PMID: 32150232 PMC7063541

[ref6] Arora D. S. Nimitvilai S. Teppen T. L. McElvain M. A. Sakharkar A. J. You C.. (2013). Hyposensitivity to gamma-aminobutyric acid in the ventral tegmental area during alcohol withdrawal: reversal by histone deacetylase inhibitors. Neuropsychopharmacology 38, 1674–1684. doi: 10.1038/npp.2013.65, PMID: 23474591 PMC3717553

[ref7] Augier E. Dulman R. S. Damadzic R. Pilling A. Hamilton J. P. Heilig M. (2017). The GABAB positive allosteric modulator ADX71441 attenuates alcohol self-administration and relapse to alcohol seeking in rats. Neuropsychopharmacology 42, 1789–1799. doi: 10.1038/npp.2017.53, PMID: 28294133 PMC5520784

[ref8] Avegno E. M. Lobell T. D. Itoga C. A. Baynes B. B. Whitaker A. M. Weera M. M.. (2018). Central amygdala circuits mediate hyperalgesia in alcohol-dependent rats. J. Neurosci. 38, 7761–7773. doi: 10.1523/JNEUROSCI.0483-18.2018, PMID: 30054393 PMC6125812

[ref9] Bahi A. (2013). Increased anxiety, voluntary alcohol consumption and ethanol-induced place preference in mice following chronic psychosocial stress. Stress 16, 441–451. doi: 10.3109/10253890.2012.754419, PMID: 23194312

[ref10] Banerjee N. (2014). Neurotransmitters in alcoholism: A review of neurobiological and genetic studies. Indian J. Hum. Genet. 20, 20–31. doi: 10.4103/0971-6866.132750, PMID: 24959010 PMC4065474

[ref11] Barchiesi R. Chanthongdee K. Domi E. Gobbo F. Coppola A. Asratian A.. (2021). Stress-induced escalation of alcohol self-administration, anxiety-like behavior, and elevated amygdala Avp expression in a susceptible subpopulation of rats. Addict. Biol. 26:e13009. doi: 10.1111/adb.13009, PMID: 33565224

[ref12] Behar K. L. Rothman D. L. Petersen K. F. Hooten M. Delaney R. Petroff O. A.. (1999). Preliminary evidence of low cortical GABA levels in localized 1H-MR spectra of alcohol-dependent and hepatic encephalopathy patients. Am. J. Psychiatr. 156, 952–954. doi: 10.1176/ajp.156.6.952, PMID: 10360140

[ref13] Belelli D. Peters J. A. Phillips G. D. Lambert J. J. (2022). The immediate and maintained effects of neurosteroids on GABAA receptors. Curr. Opin. Endocrine Metab. Res. 24:100333. doi: 10.1016/j.coemr.2022.100333, PMID: 16205363

[ref14] Berro L. F. Ruedi-Bettschen D. Cook J. E. Golani L. K. Li G. Jahan R.. (2019). GABA(A) receptor subtypes and the abuse-related effects of ethanol in rhesus monkeys: experiments with selective positive allosteric modulators. Alcohol. Clin. Exp. Res. 43, 791–802. doi: 10.1111/acer.14000, PMID: 30861153 PMC6601614

[ref15] Besheer J. Lepoutre V. Hodge C. W. (2004). GABA B receptor agonists reduce operant ethanol self-administration and enhance ethanol sedation in C57BL/6J mice. Psychopharmacology 174, 358–366. doi: 10.1007/s00213-003-1769-3, PMID: 14985930

[ref16] Bobrova N. P. Covaltchuk V. G. (1980). “The effect of ethanol and acetaldehyde on GABA transport systems and its synthesis in rat BRAIN synaptosomes” in Synaptic constituents in health and disease. eds. Brzin M. Sket D. Bachelard H. (Amsterdam: Elsevier), 109.

[ref17] Boehm S. L. II Ponomarev I. Jennings A. W. Whiting P. J. Rosahl T. W. Blednov Y. A.. (2004). γ-Aminobutyric acid A receptor subunit mutant mice: new perspectives on alcohol actions. Biochem. Pharmacol. 68, 1581–1602. doi: 10.1016/j.bcp.2004.07.023, PMID: 15451402

[ref18] Bogenschutz M. P. Bhatt S. Bohan J. Foster B. Romo P. Wilcox C. E.. (2016). Coadministration of disulfiram and lorazepam in the treatment of alcohol dependence and co-occurring anxiety disorder: an open-label pilot study. Am. J. Drug Alcohol Abuse 42, 490–499. doi: 10.3109/00952990.2016.1168430, PMID: 27184605 PMC5903270

[ref19] Bohnsack J. P. Hughes B. A. O'Buckley T. K. Edokpolor K. Besheer J. Morrow A. L. (2018). Histone deacetylases mediate GABAA receptor expression, physiology, and behavioral maladaptations in rat models of alcohol dependence. Neuropsychopharmacology 43, 1518–1529. doi: 10.1038/s41386-018-0034-8, PMID: 29520058 PMC5983537

[ref20] Bowery N. G. Hudson A. L. Price G. W. (1987). GABAA and GABAB receptor site distribution in the rat central nervous system. Neuroscience 20, 365–383. doi: 10.1016/0306-4522(87)90098-4, PMID: 3035421

[ref21] Burnette E. M. Nieto S. J. Grodin E. N. Meredith L. R. Hurley B. Miotto K.. (2022). Novel agents for the pharmacological treatment of alcohol use disorder. Drugs 82, 251–274. doi: 10.1007/s40265-021-01670-3, PMID: 35133639 PMC8888464

[ref22] Cai K. Nanga R. P. Lamprou L. Schinstine C. Elliott M. Hariharan H.. (2012). The impact of gabapentin administration on brain GABA and glutamate concentrations: a 7T ^1^H-MRS study. Neuropsychopharmacology 37, 2764–2771. doi: 10.1038/npp.2012.142, PMID: 22871916 PMC3499716

[ref23] Carpenter J. E. LaPrad D. Dayo Y. DeGrote S. Williamson K. (2018). An overview of pharmacotherapy options for alcohol use disorder. Federal Practitioner 35, 48–58. PMID: 30766325 PMC6248154

[ref24] Carroll K. M. Kiluk B. D. (2017). Cognitive behavioral interventions for alcohol and drug use disorders: through the stage model and back again. Psychol. Addict. Behav. 31:847. doi: 10.1037/adb0000311, PMID: 28857574 PMC5714654

[ref25] Carvalho A. F. Heilig M. Perez A. Probst C. Rehm J. (2019). Alcohol use disorders. Lancet 394, 781–792. doi: 10.1016/S0140-6736(19)31775-1, PMID: 31478502

[ref26] Castaldelli-Maia J. M. Bhugra D. (2022). Analysis of global prevalence of mental and substance use disorders within countries: focus on sociodemographic characteristics and income levels. Int. Rev. Psychiatry 34, 6–15. doi: 10.1080/09540261.2022.2040450, PMID: 35584016

[ref27] Ceci F. M. Francati S. Ferraguti G. Coriale G. Ciccarelli R. Minni A.. (2022). Behavioral dysregulations by chronic alcohol abuse. Motivational enhancement therapy and cognitive behavioral therapy outcomes. Riv. Psichiatr. 57, 1–9. doi: 10.1708/3749.37321, PMID: 35166724

[ref28] Chand S. P. Kuckel D. P. Huecker M. R. (2023). “Cognitive Behavior Therapy,” in StatPearls [Internet]. (Treasure Island (FL): StatPearls Publishing) Available at: https://www.ncbi.nlm.nih.gov/books/NBK470241/29261869

[ref29] Chandler C. M. Overton J. S. Rüedi-Bettschen D. Platt D. M. (2017). “GABAA Receptor Subtype Mechanisms and the Abuse-Related Effects of Ethanol: Genetic and Pharmacological Evidence,” in The Neuropharmacology of Alcohol. Handbook of Experimental Pharmacology, Eds. Grant K. Lovinger D. (Springer, Cham). 248, doi: 10.1007/164_2017_80 29204713

[ref30] Chandrasekar R. (2013). Alcohol and NMDA receptor: current research and future direction. Front. Mol. Neurosci. 6:14. doi: 10.3389/fnmol.2013.00014, PMID: 23754976 PMC3664776

[ref31] Chapp A. D. Mermelstein P. G. Thomas M. J. (2021). The ethanol metabolite acetic acid activates mouse nucleus accumbens shell medium spiny neurons. J. Neurophysiol. 125, 620–627. doi: 10.1152/jn.00659.2020, PMID: 33405999 PMC7948140

[ref32] Chen Z.-W. Bracamontes J. R. Budelier M. M. Germann A. L. Shin D. J. Kathiresan K.. (2019). Multiple functional neurosteroid binding sites on GABAA receptors. PLoS Biol. 17:e3000157. doi: 10.1371/journal.pbio.3000157, PMID: 30845142 PMC6424464

[ref33] Chiva-Blanch G. Badimon L. J. N. (2019). Benefits and risks of moderate alcohol consumption on cardiovascular disease: current findings and controversies. Nutrients 12:108. doi: 10.3390/nu12010108 31906033 PMC7020057

[ref34] Chmielewski W. X. Zink N. Chmielewski K. Y. Beste C. AK S. (2020). How high-dose alcohol intoxication affects the interplay of automatic and controlled processes. Addict. Biol. 25:e12700. doi: 10.1111/adb.12700 30561794

[ref35] Colasanti A. Searle G. E. Long C. J. Hill S. P. Reiley R. R. Quelch D.. (2012). Endogenous opioid release in the human brain reward system induced by acute amphetamine administration. Biol. Psychiatry 72, 371–377. doi: 10.1016/j.biopsych.2012.01.027, PMID: 22386378

[ref36] Colombo G. Lobina C. Maccioni P. Carai M. A. M. Lorrai I. Zaru A.. (2015). Anxiety-like behaviors at the end of the nocturnal period in sP rats with a "history" of unpredictable, limited access to alcohol. Alcohol 49, 707–712. doi: 10.1016/j.alcohol.2015.04.010, PMID: 26254964 PMC4636466

[ref37] Conte A. Attilia M. L. Gilio F. Iacovelli E. Frasca V. Marini Bettolo C.. (2008). Acute and chronic effects of ethanol on cortical excitability. Clin. Neurophysiol. 119, 667–674. doi: 10.1016/j.clinph.2007.10.021, PMID: 18083628

[ref38] Cooney G. Heydtmann M. Smith I. D. (2019). Baclofen and the alcohol withdrawal syndrome-A short review. Front. Psych. 9:773. doi: 10.3389/fpsyt.2018.00773, PMID: 30723432 PMC6349735

[ref39] Covault J. Gelernter J. Hesselbrock V. Nellissery M. Kranzler H. R. (2004). Allelic and haplotypic association of GABRA2 with alcohol dependence. American journal of medical genetics – neuropsychiatric. Genetics 129B, 104–109. doi: 10.1002/ajmg.b.30091 15274050

[ref40] Daskalakis Z. J. Möller B. Christensen B. K. Fitzgerald P. B. Gunraj C. Chen R. (2006). The effects of repetitive transcranial magnetic stimulation on cortical inhibition in healthy human subjects. Exp. Brain Res. 174, 403–412. doi: 10.1007/s00221-006-0472-0, PMID: 16683138

[ref41] Daviet R. Aydogan G. Jagannathan K. Spilka N. Koellinger P. D. Koellinger P. D.. (2022). Associations between alcohol consumption and gray and white matter volumes in the UK biobank. Nat. Commun. 13:1175. doi: 10.1038/s41467-022-28735-5, PMID: 35246521 PMC8897479

[ref42] Davis T. J. de Fiebre C. M. (2006). Alcohol's actions on neuronal nicotinic acetylcholine receptors. Alcohol Res. Health 29, 179–185. PMID: 17373406 PMC6527039

[ref43] De Beaurepaire R. (2018). A review of the potential mechanisms of action of baclofen in alcohol use disorder. Front. Psych. 9, 1–12. doi: 10.3389/fpsyt.2018.00506 PMC6232933 30459646

[ref44] de Miguel E. Vekovischeva O. Kuokkanen K. Vesajoki M. Paasikoski N. Kaskinoro J.. (2019). GABAB receptor positive allosteric modulators with different efficacies affect neuroadaptation to and self-administration of alcohol and cocaine. Addict. Biol. 24, 1191–1203. doi: 10.1111/adb.12688, PMID: 30421860

[ref45] De Witte P. (2004). Imbalance between neuroexcitatory and neuroinhibitory amino acids causes craving for ethanol. Addict. Behav. 29, 1325–1339. doi: 10.1016/j.addbeh.2004.06.020, PMID: 15345268

[ref46] Dhaliwal J. S. Rosani A. Saadabadi A. (2022). “Diazepam,” in StatPearls [internet]. Treasure Island (FL): StatPearls Publishing. Available at: https://www.ncbi.nlm.nih.gov/books/NBK537022/30725707

[ref47] Dodd P. R. Tracey Buckley S. Eckert A. L. Foley P. F. Innes D. J. (2006). Genes and gene expression in the brains of human alcoholics. Ann. NY Acad. Sci. 1074, 104–115. doi: 10.1196/annals.1369.010, PMID: 17105908

[ref48] Engin E. (2022). GABAA receptor subtypes and benzodiazepine use, misuse, and abuse. Front. Psych. 13:2931. doi: 10.3389/fpsyt.2022.1060949 PMC9879605 36713896

[ref80] Enna S. J. (2007). The GABA receptors. Humana Press.

[ref49] Enoch M. A. (2008). The role of GABA(A) receptors in the development of alcoholism. Pharmacol. Biochem. Behav. 90, 95–104. doi: 10.1016/j.pbb.2008.03.007, PMID: 18440057 PMC2577853

[ref50] Fariba K. A. Saadabadi A. (2022). Topiramate. StatPearls: Tampa, FL. 32119417

[ref51] Ferraguti G. Pascale E. Lucarelli M. (2015). Alcohol addiction: a molecular biology perspective. Curr. Med. Chem. 22, 670–684. doi: 10.2174/0929867321666141229103158, PMID: 25544474

[ref52] Foo J. C. Vengeliene V. Noori H. R. Yamaguchi I. Morita K. Nakamura T.. (2019). Drinking levels and profiles of alcohol addicted rats predict response to Nalmefene. Front. Pharmacol. 10:471. doi: 10.3389/fphar.2019.00471, PMID: 31133855 PMC6513880

[ref53] Franck J. Jayaram-Lindström N. (2013). Pharmacotherapy for alcohol dependence: status of current treatments. Curr. Opin. Neurobiol. 23, 692–699. doi: 10.1016/j.conb.2013.05.005, PMID: 23810221

[ref54] Fritschy J. M. Mohler H. (1995). GABAA-receptor heterogeneity in the adult rat brain: differential regional and cellular distribution of seven major subunits. J. Comp. Neurol. 359, 154–194. doi: 10.1002/cne.903590111, PMID: 8557845

[ref55] Gallager D. Lakoski J. Gonsalves S. Rauch S. J. N. (1984). Chronic benzodiazepine treatment decreases postsynaptic GABA sensitivity. Nature 308, 74–77. 6322004 10.1038/308074a0

[ref56] Garbutt J. C. (2019). Approval of baclofen for alcohol use disorders in France: A perspective from the United States. Alcohol Alcohol. 55:48. doi: 10.1093/alcalc/agz084 31761948

[ref57] Gatta E. Camussi D. Auta J. Guidotti A. Pandey S. C. (2022). Neurosteroids (allopregnanolone) and alcohol use disorder: from mechanisms to potential pharmacotherapy. Pharmacol. Ther. 240:108299. doi: 10.1016/j.pharmthera.2022.108299, PMID: 36323379 PMC9810076

[ref58] Ghiasi N. Bhansali R. K. Marwaha R. (2023). “Lorazepam” in StatPearls [Internet], (Treasure Island (FL): StatPearls Publishing). Available at: https://www.ncbi.nlm.nih.gov/books/NBK532890/30422485

[ref59] Ghit A. Assal D. Al-Shami A. S. Hussein D. E. E. (2021). GABA(A) receptors: structure, function, pharmacology, and related disorders. J. Genet. Eng. Biotechnol. 19:123. doi: 10.1186/s43141-021-00224-0, PMID: 34417930 PMC8380214

[ref60] Gilman J. M. Ramchandani V. A. Davis M. B. Bjork J. M. DWJJoN H. (2008). Why we like to drink: a functional magnetic resonance imaging study of the rewarding and anxiolytic effects of alcohol. J. Neurosci. 28, 4583–4591. doi: 10.1523/JNEUROSCI.0086-08.2008 18448634 PMC2730732

[ref61] Gil-Mohapel J. Bianco C. D. Cesconetto P. A. Zamoner A. Brocardo P. S. (2019). “Chapter 51 – ethanol exposure during development, and brain oxidative stress” in Neuroscience of alcohol. ed. Preedy V. R. (Cambridge, MA: Academic Press), 493–503.

[ref62] Giuliano C. Peña-Oliver Y. Goodlett C. R. Cardinal R. N. Robbins T. W. Bullmore E. T.. (2018). Evidence for a Long-lasting compulsive alcohol seeking phenotype in rats. Neuropsychopharmacology 43, 728–738. doi: 10.1038/npp.2017.105, PMID: 28553834 PMC5809777

[ref63] Goeders N. E. Guerin G. F. (2008). Effects of the combination of metyrapone and oxazepam on cocaine and food self-administration in rats. Pharmacol. Biochem. Behav. 91, 181–189. doi: 10.1016/j.pbb.2008.07.005, PMID: 18692521 PMC2564858

[ref64] Gomes T. Juurlink D. N. Antoniou T. Mamdani M. M. Paterson J. M. van den Brink W. (2017). Gabapentin, opioids, and the risk of opioid-related death: A population-based nested case-control study. PLoS Med. 14:e1002396. doi: 10.1371/journal.pmed.1002396, PMID: 28972983 PMC5626029

[ref65] Grant K. A. Lovinger D. M. (2018). The neuropharmacology of alcohol. New York: Springer.

[ref66] Haile C. N. Carper B. A. Nolen T. L. Kosten T. A. (2021). The GABA(B) receptor positive allosteric modulator ASP8062 reduces operant alcohol self-administration in male and female Sprague Dawley rats. Psychopharmacology 238, 2587–2600. doi: 10.1007/s00213-021-05881-0, PMID: 34228136

[ref67] Harel M. Perini I. Kämpe R. Alyagon U. Shalev H.. (2022). Repetitive transcranial magnetic stimulation in alcohol dependence: A randomized, double-blind, sham-controlled proof-of-concept trial targeting the medial prefrontal and anterior cingulate cortices. Biol. Psychiatry 91, 1061–1069. doi: 10.1016/j.biopsych.2021.11.020, PMID: 35067356

[ref68] Heinz A. Beck A. Grüsser S. M. Grace A. A. Wrase J. (2009). Identifying the neural circuitry of alcohol craving and relapse vulnerability. Addict. Biol. 14, 108–118. doi: 10.1111/j.1369-1600.2008.00136.x, PMID: 18855799 PMC2879014

[ref69] Herman M. A. Contet C. Roberto M. (2016). A functional switch in tonic GABA currents alters the output of central amygdala corticotropin releasing factor receptor-1 neurons following chronic ethanol exposure. J. Neurosci. 36, 10729–10741. doi: 10.1523/JNEUROSCI.1267-16.2016 27798128 PMC5083004

[ref70] Herman M. A. Roberto M. (2016). Cell-type-specific tonic GABA signaling in the rat central amygdala is selectively altered by acute and chronic ethanol. J. Neurosci. 21, 72–86. doi: 10.1111/adb.12181 PMC4345144 25170988

[ref71] Higgins-Biddle J. C. Babor T. F. (2018). A review of the alcohol use disorders identification test (AUDIT), AUDIT-C, and USAUDIT for screening in the United States: past issues and future directions. Am. J. Drug Alcohol Abuse 44, 578–586. doi: 10.1080/00952990.2018.1456545, PMID: 29723083 PMC6217805

[ref72] Hodgins D. C. Ching L. E. McEwen J. (2009). Strength of commitment language in motivational interviewing and gambling outcomes. Psychol. Addict. Behav. 23, 122–130. doi: 10.1037/a0013010, PMID: 19290696

[ref73] Holford N. H. (1987). Clinical pharmacokinetics of ethanol. Clin. Pharmacokinet 13, 273–292. doi: 10.2165/00003088-198713050-00001 3319346

[ref74] Holtyn A. F. Weerts E. M. (2022). GABA(B) receptors and alcohol use disorders: preclinical studies. Curr. Top. Behav. Neurosci. 52, 157–194. doi: 10.1007/7854_2020_178, PMID: 32808090

[ref75] Hoyumpa A. M. Jr. (1984). Alcohol interactions with benzodiazepines and cocaine. Adv. Alcohol Subst. Abuse 3, 21–34. doi: 10.1300/J251v03n04_03 6391107

[ref76] Hughes B. A. Bohnsack J. P. O'Buckley T. K. Herman M. A. Morrow A. L. (2019). Chronic ethanol exposure and withdrawal impair synaptic GABA(A) receptor-mediated neurotransmission in deep-layer prefrontal cortex. Alcohol. Clin. Exp. Res. 43, 822–832. doi: 10.1111/acer.14015, PMID: 30860602 PMC6502689

[ref77] Hwa L. S. Kalinichev M. Haddouk H. Poli S. Miczek K. A. (2014). Reduction of excessive alcohol drinking by a novel GABAB receptor positive allosteric modulator ADX71441 in mice. Psychopharmacology 231, 333–343. doi: 10.1007/s00213-013-3245-z, PMID: 23975038 PMC3947346

[ref78] Ito M. Spence A. Blauwet M. B. Heo N. Goldwater R. Maruff P.. (2022). A phase 1 study to assess potential interaction between ASP8062 and alcohol in healthy adult subjects. J. Psychopharmacol. 36, 756–767. doi: 10.1177/02698811211058967, PMID: 34994232

[ref79] Janmohamed M. Brodie M. J. Kwan P. (2020). Pharmacoresistance–epidemiology, mechanisms, and impact on epilepsy treatment. Neuropharmacology 168:107790. doi: 10.1016/j.neuropharm.2019.107790, PMID: 31560910

[ref81] Jesse S. Bråthen G. Ferrara M. Keindl M. Ben-Menachem E. Tanasescum R.. (2017). Alcohol withdrawal syndrome: mechanisms, manifestations, and management. Acta Neurol. Scand. 135, 4–16. doi: 10.1111/ane.12671, PMID: 27586815 PMC6084325

[ref82] Johannessen C. U. Johannessen S. I. (2003). Valproate: past, present, and future. CNS Drug Rev. 9, 199–216. doi: 10.1111/j.1527-3458.2003.tb00249.x, PMID: 12847559 PMC6741662

[ref83] Jonas D. E. Amick H. R. Feltner C. Bobashev G. Thomas K. Wines R.. (2014). Pharmacotherapy for adults with alcohol use disorders in outpatient settings: A systematic review and meta-analysis. JAMA 311, 1889–1900. doi: 10.1001/jama.2014.3628, PMID: 24825644

[ref84] June H. L. Harvey S. C. Foster K. L. McKay P. F. Cummings R. Garcia M.. (2001). GABA_A_receptors containing α5 subunits in the CA1 and CA3 hippocampal Fields regulate ethanol-motivated Behaviors: an extended ethanol reward circuitry. J. Neurosci. 21, 2166–2177. doi: 10.1523/JNEUROSCI.21-06-02166.2001, PMID: 11245701 PMC6762602

[ref85] Kaarre O. Kallioniemi E. Könönen M. Tolmunen T. Kekkonen V. Kivimäki P.. (2018). Heavy alcohol use in adolescence is associated with altered cortical activity: a combined TMS-EEG study. Addict. Biol. 23, 268–280. doi: 10.1111/adb.12486, PMID: 28008690

[ref86] Kalk N. J. Lingford-Hughes A. R. (2014). The clinical pharmacology of acamprosate. Br. J. Clin. Pharmacol. 77, 315–323. doi: 10.1111/bcp.12070, PMID: 23278595 PMC4014018

[ref87] Kirkland A. E. Browning B. D. Green R. Leggio L. Meyerhoff D. J. Squeglia L. M. (2022). Brain metabolite alterations related to alcohol use: a meta-analysis of proton magnetic resonance spectroscopy studies. Mol. Psychiatry 27, 3223–3236. doi: 10.1038/s41380-022-01594-8, PMID: 35508628 PMC10578135

[ref88] Kisby B. R. Farris S. P. McManus M. M. Varodayan F. P. Roberto M. Harris R. A.. (2021). Alcohol dependence in rats is associated with global changes in gene expression in the central amygdala. Brain Sci. 11:1149. doi: 10.3390/brainsci11091149, PMID: 34573170 PMC8468792

[ref89] Knapp D. J. Overstreet D. H. Breese G. R. (2007). Baclofen blocks expression and sensitization of anxiety-like behavior in an animal model of repeated stress and ethanol withdrawal. Alcohol. Clin. Exp. Res. 31, 582–595. doi: 10.1111/j.1530-0277.2007.00342.x, PMID: 17374037 PMC2864137

[ref90] Koob G. F. Volkow N. D. (2016). Neurobiology of addiction: a neurocircuitry analysis. Lancet Psychiatry 3, 760–773. doi: 10.1016/S2215-0366(16)00104-8, PMID: 27475769 PMC6135092

[ref91] Kranzler H. R. Covault J. Feinn R. Armeli S. Tennen H. Arias A. J.. (2014). Topiramate treatment for heavy drinkers: moderation by a GRIK1 polymorphism. Am. J. Psychiatry 171, 445–452. doi: 10.1176/appi.ajp.2013.13081014, PMID: 24525690 PMC3997125

[ref92] Kranzler H. R. Soyka M. (2018). Diagnosis and pharmacotherapy of alcohol use disorder: a review. JAMA 320, 815–824. doi: 10.1001/jama.2018.11406, PMID: 30167705 PMC7391072

[ref93] Kruse L. C. Linsenbardt D. N. Boehm S. L. (2012). Positive allosteric modulation of the GABA B receptor by GS39783 attenuates the locomotor stimulant actions of ethanol and potentiates the induction of locomotor sensitization. Alcohol 46, 455–462. doi: 10.1016/j.alcohol.2012.03.004, PMID: 22560291 PMC3389305

[ref94] Kumar S. Porcu P. Werner D. F. Matthews D. B. Diaz-Granados J. L. Helfand R. S.. (2009). The role of GABAA receptors in the acute and chronic effects of ethanol: a decade of progress. Psychopharmacology 205, 529–564. doi: 10.1007/s00213-009-1562-z 19455309 PMC2814770

[ref95] Laniepce A. Cabé N. André C. Bertran F. Boudehent C. Lahbairi N.. (2020). The effect of alcohol withdrawal syndrome severity on sleep, brain and cognition. Brain. Communications 2:fcaa123. doi: 10.1093/braincomms/fcaa123, PMID: 33543128 PMC7846181

[ref96] Larsson S. C. Burgess S. Mason A. M. Michaëlsson K. (2020). Alcohol consumption and cardiovascular disease: A mendelian randomization study. Circ. Genom. Precis. Med. 13:e002814. doi: 10.1161/CIRCGEN.119.002814, PMID: 32367730 PMC7299220

[ref97] Larsson A. Edström L. Svensson L. Söderpalm B. Engel J. A. (2005). Voluntary ethanol intake increases extracellular acetylcholine levels in the ventral tegmental area in the rat. Alcohol Alcohol. 40, 349–358. doi: 10.1093/alcalc/agh180, PMID: 16043436

[ref98] Leggio G. M. Di Marco R. Gulisano W. D’Ascenzo M. Torrisi S. A. Geraci F.. (2019). Dopaminergic-GABAergic interplay and alcohol binge drinking. Pharmacol. Res. 141, 384–391. doi: 10.1016/j.phrs.2019.01.022, PMID: 30648615

[ref99] Li J. Bian W. Dave V. Ye J.-H. (2011). Blockade of GABAA receptors in the paraventricular nucleus of the hypothalamus attenuates voluntary ethanol intake and activates the hypothalamic–pituitary–adrenocortical axis. Addict. Biol. 16, 600–614. doi: 10.1111/j.1369-1600.2011.00344.x 21762292

[ref100] Liang J. Cagetti E. Olsen R. W. IJJoP S. Therapeutics E. (2004). Altered pharmacology of synaptic and extrasynaptic GABAA receptors on CA1 hippocampal neurons is consistent with subunit changes in a model of alcohol withdrawal and dependence. J. Pharmacol. Exp. Ther. 310, 1234–1245. doi: 10.1124/jpet.104.067983 15126642

[ref101] Liang J. Olsen R. W. (2014). Alcohol use disorders and current pharmacological therapies: the role of GABA(A) receptors. Acta Pharmacol. Sin. 35, 981–993. doi: 10.1038/aps.2014.50, PMID: 25066321 PMC4125717

[ref102] Linsenbardt D. N. Boehm Ii S. L. (2014). Alterations in the rate of binge ethanol consumption: implications for preclinical studies in mice. Addict. Biol. 19, 812–825. doi: 10.1111/adb.12052, PMID: 23742054 PMC3775999

[ref103] Littleton J. (1998). Neurochemical mechanisms underlying alcohol withdrawal. Alcohol. Health Res. World 22, 13–24. PMID: 15706728 PMC6761820

[ref104] Logge W. B. Morley K. C. Haber P. S. (2022). GABA(B) receptors and alcohol use disorders: clinical studies. Curr. Top. Behav. Neurosci. 52, 195–212. doi: 10.1007/7854_2020_182, PMID: 33580440

[ref105] Loi B. Maccioni P. Lobina C. Carai M. A. M. Gessa G. L. Thomas A. W.. (2013). Reduction of alcohol intake by the positive allosteric modulator of the GABAB receptor, rac-BHFF, in alcohol-preferring rats. Alcohol 47, 69–73. doi: 10.1016/j.alcohol.2012.11.002, PMID: 23218664

[ref106] López-Pelayo H. Zuluaga P. Caballeria E. Van den Brink W. Mann K. Gual A. (2020). Safety of nalmefene for the treatment of alcohol use disorder: an update. Expert Opin. Drug Saf. 19, 9–17. doi: 10.1080/14740338.2020.1707802, PMID: 31868031

[ref107] Lorrai I. Contini A. Gessa G. L. Mugnaini C. Corelli F. Colombo G.. (2019). Operant, oral alcohol self-administration: sex differences in Sardinian alcohol-preferring rats. Alcohol 79, 147–162. doi: 10.1016/j.alcohol.2019.04.003, PMID: 31029630

[ref108] Lorrai I. Shankula C. Gaytan J. M. Kawamura T. Maccioni P. Mugnaini C.. (2022). Reducing effect of the novel positive allosteric modulator of the GABA(B) receptor, COR659, on binge-like alcohol drinking in male mice and rats. Psychopharmacology 239, 201–213. doi: 10.1007/s00213-021-06022-3, PMID: 34812900

[ref109] Lovinger D. M. (1997). Serotonin's role in alcohol's effects on the brain. Alcohol Health Res. World 21, 114–120. PMID: 15704346 PMC6826824

[ref110] Ma H. Zhu G. (2014). The dopamine system and alcohol dependence. Shanghai Arch. Psychiatry 26, 61–68. doi: 10.3969/j.issn.1002-0829.2014.02.002, PMID: 25092951 PMC4120286

[ref111] MacCioni P. Carai M. A. M. Kaupmann K. Guery S. Froestl W. Leite-Morrism K.. (2009). Reduction of alcohol's reinforcing and motivational properties by the positive allosteric modulator of the GABAB receptor, BHF177, in alcohol-preferring rats. Alcohol. Clin. Exp. Res. 33, 1749–1756. doi: 10.1111/j.1530-0277.2009.01012.x, PMID: 19572981

[ref112] Maccioni P. Colombo G. (2019). Potential of GABA(B) receptor positive allosteric modulators in the treatment of alcohol use disorder. CNS Drugs 33, 107–123. doi: 10.1007/s40263-018-0596-3, PMID: 30604283

[ref113] Maccioni P. Colombo G. Lorrai I. Zaru A. Carai M. A. M. Gessa G. L.. (2017). Suppressing effect of COR659 on alcohol, sucrose, and chocolate self-administration in rats: involvement of the GABAB and cannabinoid CB1 receptors. Psychopharmacology 234, 2525–2543. doi: 10.1007/s00213-017-4644-3, PMID: 28536867

[ref114] Maccioni P. Kaczanowska K. McDonald P. Colombo G. (2022). Development of partial tolerance to the suppressing effect of the positive allosteric modulator of the GABAB receptor, KK-92A, on alcohol self-Administration in Rats. Alcohol Alcohol. 57, 706–711. doi: 10.1093/alcalc/agac026, PMID: 35589119

[ref115] Maccioni P. Lorrai I. Contini A. Leite-Morris K. Colombo G. (2018). Microinjection of baclofen and CGP7930 into the ventral tegmental area suppresses alcohol self-administration in alcohol-preferring rats. Neuropharmacology 136, 146–158. doi: 10.1016/j.neuropharm.2017.10.012, PMID: 29050951

[ref116] Maccioni P. Vargiolu D. Thomas A. W. Malherbe P. Mugnaini C. Corelli F.. (2015). Inhibition of alcohol self-administration by positive allosteric modulators of the GABAB receptor in rats: lack of tolerance and potentiation of baclofen. Psychopharmacology 232, 1831–1841. doi: 10.1007/s00213-014-3815-8, PMID: 25420609

[ref117] Mallard T. T. Ashenhurst J. R. Harden K. P. Fromme K. (2018). GABRA2, alcohol, and illicit drug use: an event-level model of genetic risk for polysubstance use. J. Abnorm. Psychol. 127, 190–201. doi: 10.1037/abn0000333, PMID: 29528673 PMC5851473

[ref118] Manhapra A. Chakraborty A. Arias A. J. (2019). Topiramate pharmacotherapy for alcohol use disorder and other addictions: A narrative review. J. Addict. Med. 13, 7–22. doi: 10.1097/ADM.0000000000000443, PMID: 30096077

[ref119] Marik P. E. (2004). Propofol: therapeutic indications and side-effects. Curr. Pharm. Des. 10, 3639–3649. doi: 10.2174/1381612043382846, PMID: 15579060

[ref120] Marinkovic K. Alderson Myers A. B. Arienzo D. Sereno M. I. Mason G. F. (2022). Cortical GABA levels are reduced in young adult binge drinkers: association with recent alcohol consumption and sex. NeuroImage 35:103091. doi: 10.1016/j.nicl.2022.103091, PMID: 35753236 PMC9240858

[ref121] Mason B. J. (2017). Emerging pharmacotherapies for alcohol use disorder. Neuropharmacology 122, 244–253. doi: 10.1016/j.neuropharm.2017.04.032, PMID: 28454983 PMC5643030

[ref122] McCaul M. E. Hutton H. E. Stephens M. A. C. Xu X. Wand G. S. (2017). Anxiety, anxiety sensitivity, and perceived stress as predictors of recent drinking, alcohol craving, and social stress response in heavy drinkers. Alcohol. Clin. Exp. Res. 41, 836–845. doi: 10.1111/acer.13350, PMID: 28281290 PMC5388456

[ref123] McCunn P. Chen X. Gimi B. Green A. I. Khokhar J. Y. (2022). Glutamine and GABA alterations in cingulate cortex may underlie alcohol drinking in a rat model of co-occurring alcohol use disorder and schizophrenia: an 1H-MRS study. Schizophrenia 8:67. doi: 10.1038/s41537-022-00272-6, PMID: 35999232 PMC9399110

[ref124] Melis M. Enrico P. Peana A. T. Diana M. (2007). Acetaldehyde mediates alcohol activation of the mesolimbic dopamine system. Eur. J. Neurosci. 26, 2824–2833. doi: 10.1111/j.1460-9568.2007.05887.x, PMID: 18001279

[ref125] Meyer E. M. Long V. Fanselow M. S. Spigelman I. (2013). Stress increases voluntary alcohol intake, but does not alter established drinking habits in a rat model of posttraumatic stress disorder. Alcohol. Clin. Exp. Res. 37, 566–574. doi: 10.1111/acer.12012, PMID: 23126586 PMC3567303

[ref126] Mira R. G. Tapia-Rojas C. Pérez M. J. Jara C. Vergara E. H.. (2019). Alcohol impairs hippocampal function: from NMDA receptor synaptic transmission to mitochondrial function. Drug Alcohol Depend. 205:107628. doi: 10.1016/j.drugalcdep.2019.107628, PMID: 31683244

[ref127] Mirijello A. Caputo F. Vassallo G. Rolland B. Tarli C. Gasbarrini A.. (2015). GABAB agonists for the treatment of alcohol use disorder. Curr. Pharm. Des. 21, 3367–3372. doi: 10.2174/1381612821666150619091858, PMID: 26088121

[ref128] Mishra B. R. Nizamie S. H. Das B. Praharaj S. K. (2010). Efficacy of repetitive transcranial magnetic stimulation in alcohol dependence: a sham-controlled study. Addiction 105, 49–55. doi: 10.1111/j.1360-0443.2009.02777.x, PMID: 20078462

[ref129] Mitchell J. M. O'Neil J. P. Janabi M. Marks S. M. Jagust W. J. Fields H. L. (2012). Alcohol consumption induces endogenous opioid release in the human orbitofrontal cortex and nucleus accumbens. Sci. Transl. Med. 4:116ra6. doi: 10.1126/scitranslmed.3002902, PMID: 22238334

[ref130] Modesto-Lowe V. Barron G. C. Aronow B. Chaplin M. (2019). Gabapentin for alcohol use disorder: A good option, or cause for concern? Cleve. Clin. J. Med. 86, 815–823. doi: 10.3949/ccjm.86a.18128, PMID: 31821139

[ref131] Mohammadi B. Krampfl K. Petri S. Bogdanova D. Kossev A. Bufler J.. (2006). Selective and nonselective benzodiazepine agonists have different effects on motor cortex excitability. Muscle Nerve 33, 778–784. doi: 10.1002/mus.20531, PMID: 16598788

[ref132] Mon A. Durazzo T. C. Meyerhoff D. J. (2012). Glutamate, GABA, and other cortical metabolite concentrations during early abstinence from alcohol and their associations with neurocognitive changes. Drug Alcohol Depend. 125, 27–36. doi: 10.1016/j.drugalcdep.2012.03.012, PMID: 22503310 PMC3419314

[ref133] Morrow A. L. Boero G. Porcu P. (2020). A rationale for allopregnanolone treatment of alcohol use disorders: basic and clinical studies. Alcohol. Clin. Exp. Res. 44, 320–339. doi: 10.1111/acer.14253, PMID: 31782169 PMC7018555

[ref134] Mounier N. M. Wahdan S. A. Gad A. M. Azab S. S. (2021). Role of inflammatory, oxidative, and ER stress signaling in the neuroprotective effect of atorvastatin against doxorubicin-induced cognitive impairment in rats. Naunyn Schmiedebergs Arch. Pharmacol. 394, 1537–1551. doi: 10.1007/s00210-021-02081-7 33755739

[ref135] Möykkynen T. Korpi E. R. (2012). Acute effects of ethanol on glutamate receptors. Basic Clin. Pharmacol. Toxicol. 111, 4–13. doi: 10.1111/j.1742-7843.2012.00879.x, PMID: 22429661

[ref136] Naim-Feil J. Bradshaw J. L. Rogasch N. C. Daskalakis Z. J. Sheppard D. M. Lubman D. I.. (2016). Cortical inhibition within motor and frontal regions in alcohol dependence post-detoxification: A pilot TMS-EEG study. World J. Biol. Psychiatry 17, 547–556. doi: 10.3109/15622975.2015.1066512, PMID: 26243644

[ref137] Nardone R. Bergmann J. Kronbichler M. Caleri F. Lochner P. Tezzon F.. (2010). Altered motor cortex excitability to magnetic stimulation in alcohol withdrawal syndrome. Alcohol. Clin. Exp. Res. 34, 628–632. doi: 10.1111/j.1530-0277.2009.01131.x, PMID: 20102563

[ref138] Nelson A. C. Kehoe J. Sankoff J. Mintzer D. Taub J. Kaucher K. A. (2019). Benzodiazepines vs barbiturates for alcohol withdrawal: analysis of 3 different treatment protocols. Am. J. Emerg. Med. 37, 733–736. doi: 10.1016/j.ajem.2019.01.002, PMID: 30685075

[ref139] Newman R. K. Gallagher M. A. S. Gomez A. E. (2023). “Alcohol withdrawal,” in StatPearls [Internet]. Treasure Island (FL): StatPearls Publishing. Available at: https://www.ncbi.nlm.nih.gov/books/NBK441882/

[ref140] NIAAA (2018). Drinking patterns and their definitions. Alcohol Res. 39, 17–18. Available at: https://arcr.niaaa.nih.gov/volume/39/1/drinking-patterns-and-their-definitions PMID: 30557143 10.35946/arcr.v39.1.03 PMC6104961

[ref141] NIAAA. (2020). Understanding Alcohol Use Disorder. Available at: https://www.niaaa.nih.gov/publications/brochures-and-fact-sheets/understanding-alcohol-use-disorder

[ref143] Olsen R. W. Liang J. (2017). Role of GABA(A) receptors in alcohol use disorders suggested by chronic intermittent ethanol (CIE) rodent model. Mol. Brain 10:45. doi: 10.1186/s13041-017-0325-8, PMID: 28931433 PMC5605989

[ref144] Orrù A. Lai P. Lobina C. Maccioni P. Piras P.. (2005). Reducing effect of the positive allosteric modulators of the GABA B receptor, CGP7930 and GS39783, on alcohol intake in alcohol-preferring rats. Eur. J. Pharmacol. 525, 105–111. doi: 10.1016/j.ejphar.2005.10.005, PMID: 16289452

[ref145] Paille F. Martini H. (2014). Nalmefene: a new approach to the treatment of alcohol dependence. Subst. Abus. Rehabil. 5, 87–94. doi: 10.2147/SAR.S45666, PMID: 25187751 PMC4133028

[ref146] Pandey S. C. Sakharkar A. J. Tang L. Zhang H. (2015). Potential role of adolescent alcohol exposure-induced amygdaloid histone modifications in anxiety and alcohol intake during adulthood. Neurobiol. Dis. 82, 607–619. doi: 10.1016/j.nbd.2015.03.019, PMID: 25814047 PMC4581895

[ref147] Paparrigopoulos T. Tzavellas E. Karaiskos D. Kourlaba G. Liappas I. (2011). Treatment of alcohol dependence with low-dose topiramate: an open-label controlled study. BMC Psychiatry 11:41. doi: 10.1186/1471-244X-11-41, PMID: 21401921 PMC3062593

[ref148] Paradis C. Butt P. Shield K. Poole N. Wells S.. (2022). Update of Canada’s low-risk alcohol drinking guidelines: Final report for public consultation. Ottawa, ON: Canadian Centre on Substance Use and Addiction.

[ref149] Patatanian E. Nguyen D. R. (2022). Brexanolone: A novel drug for the treatment of postpartum depression. J. Pharm. Pract. 35, 431–436. doi: 10.1177/0897190020979627, PMID: 33302791

[ref150] Peana A. T. Sánchez-Catalán M. J. Hipólito L. Rosas M. Porru S.. (2017). Mystic acetaldehyde: the never-ending story on alcoholism. Front. Behav. Neurosci. 11:81. doi: 10.3389/fnbeh.2017.00081, PMID: 28553209 PMC5425597

[ref151] Pérez-Ramírez Ú. López-Madrona V. J. Pérez-Segura A. Pallarés V. Moreno A.. (2022). Brain network allostasis after chronic alcohol drinking is characterized by functional dedifferentiation and narrowing. J. Neurosci. 42, 4401–4413. doi: 10.1523/JNEUROSCI.0389-21.2022, PMID: 35437279 PMC9145238

[ref152] Pinard A. Seddik R. Bettler B. (2010). GABAB receptors: physiological functions and mechanisms of diversity. Adv. Pharmacol. 58, 231–255. doi: 10.1016/S1054-3589(10)58010-4, PMID: 20655485

[ref153] Pinna G. Almeida F. B. Davis J. M. (2022). Allopregnanolone in Postpartum Depression. Front. Glob. Womens Health 3:823616. doi: 10.3389/fgwh.2022.823616, PMID: 35558166 PMC9088875

[ref154] Pleil K. E. Lowery-Gionta E. G. Crowley N. A. Li C. Marcinkiewcz C. A.. (2015). Effects of chronic ethanol exposure on neuronal function in the prefrontal cortex and extended amygdala. Neuropharmacology 99, 735–749. doi: 10.1016/j.neuropharm.2015.06.017 26188147 PMC4781662

[ref155] Prevot T. Sibille E. (2021). Altered GABA-mediated information processing and cognitive dysfunctions in depression and other brain disorders. Mol. Psychiatry 26, 151–167. doi: 10.1038/s41380-020-0727-3, PMID: 32346158

[ref156] Prisciandaro J. J. Schacht J. P. Prescot A. P. Renshaw P. F. Brown T. R. Anton R. F. (2019). Brain glutamate, GABA, and glutamine levels and associations with recent drinking in treatment-Naïve individuals with alcohol use disorder versus light drinkers. Alcohol 43, 221–226. doi: 10.1111/acer.13931, PMID: 30537347 PMC6538297

[ref157] Quertemont E. Grant K. A. Correa M. Arizzi M. N. Salamone J. D.. (2005). The role of acetaldehyde in the central effects of ethanol. Alcohol. Clin. Exp. Res. 29, 221–234. doi: 10.1097/01.ALC.0000156185.39073.D2, PMID: 15714045

[ref158] Rehm J. Gmel G. E. Sr. Gmel G. Hasan O. S. M. Imtiaz S.. (2017). The relationship between different dimensions of alcohol use and the burden of disease-an update. Addiction 112, 968–1001. doi: 10.1111/add.13757, PMID: 28220587 PMC5434904

[ref159] Rolland B. Simon N. Franchitto N. Aubin H. J. (2020). France Grants an approval to baclofen for alcohol dependence. Alcohol Alcohol. 55, 44–45. doi: 10.1093/alcalc/agz082, PMID: 31761949

[ref160] Romeo E. Brancati A. De Lorenzo A. Fucci P. Furnari C.. (1996). Marked decrease of plasma neuroactive steroids during alcohol withdrawal. Clin. Neuropharmacol. 19, 366–369. doi: 10.1097/00002826-199619040-00011, PMID: 8829001

[ref161] Romito J. W. Turner E. R. Rosener J. A. Coldiron L. Udipi A.. (2021). Baclofen therapeutics, toxicity, and withdrawal: a narrative review. SAGE Open Med. 9:205031212110221. doi: 10.1177/20503121211022197, PMID: 34158937 PMC8182184

[ref162] Rüedi-Bettschen D. Rowlett J. K. Rallapalli S. Clayton T. Cook J. M. Platt D. M. (2013). Modulation of α5 subunit-containing GABAA receptors alters alcohol drinking by rhesus monkeys. Alcohol. Clin. Exp. Res. 37, 624–634. doi: 10.1111/acer.12018, PMID: 23126673 PMC3951841

[ref163] Sanchez-Catalan M. J. Kaufling J. Georges F. Veinante P. Barrot M. (2014). The antero-posterior heterogeneity of the ventral tegmental area. Neuroscience 282, 198–216. doi: 10.1016/j.neuroscience.2014.09.025, PMID: 25241061

[ref164] Sarasa S. B. Mahendran R. Muthusamy G. Thankappan B. Selta D. R. F. Angayarkanni J. J. C. (2020). A brief review on the non-protein amino acid, gamma-amino butyric acid (GABA): its production and role in microbes. Curr. Microbiol. 77, 534–544. doi: 10.1007/s00284-019-01839-w 31844936

[ref165] Saunders J. B. Degenhardt L. Reed G. M. Poznyak V. (2019). Alcohol use disorders in ICD-11: past, present, and future. Alcohol. Clin. Exp. Res. 43, 1617–1631. doi: 10.1111/acer.14128 31194891

[ref166] Savin Z. Kivity S. Yonath H. Yehuda S. (2018). Smoking and the intestinal microbiome. Arch. Microbiol. 200, 677–684. doi: 10.1007/s00203-018-1506-2, PMID: 29626219

[ref167] Schmidt K. J. Doshi M. R. Holzhausen J. M. Natavio A. Cadiz M. Winegardner J. E. (2016). Treatment of severe alcohol withdrawal. Ann. Pharmacother. 50, 389–401. doi: 10.1177/1060028016629161, PMID: 26861990

[ref168] Shyu C. Chavez S. Boileau I. Le Foll B. (2022). Quantifying GABA in addiction: A review of proton magnetic resonance spectroscopy studies. Brain Sci. 12:918. doi: 10.3390/brainsci12070918, PMID: 35884725 PMC9316447

[ref169] Singh D. Saadabadi A. (2023). “Naltrexone,” in StatPearls [Internet]. Treasure Island, FL: StatPearls Publishing. Available at: https://www.ncbi.nlm.nih.gov/books/NBK534811/

[ref170] Sloan M. E. Werner R. B. Yarnell-Macgrory S. Petrakis I. (2020). Alcohol, pp. 121–137: New York: Springer International Publishing

[ref171] Solanki N. Abijo T. Galvao C. Darius P. Blum K. Gondré-Lewis M. C. (2020). Administration of a putative pro-dopamine regulator, a neuronutrient, mitigates alcohol intake in alcohol-preferring rats. Behav. Brain Res. 385:112563. doi: 10.1016/j.bbr.2020.112563, PMID: 32070691 PMC7244251

[ref172] Sorkhou M. Stogios N. Sayrafizadeh N. Hahn M. K. Agarwal S. M. George T. P. (2022). Non-invasive neuromodulation of dorsolateral prefrontal cortex to reduce craving in alcohol use disorder: A meta-analysis. Drug Alcohol Depend. Rep. 4:100076. doi: 10.1016/j.dadr.2022.100076, PMID: 36846579 PMC9948891

[ref173] Soyka M. Preuss U. W. Hesselbrock V. Zill P. Koller G. Bondy B. (2008). GABA-A2 receptor subunit gene (GABRA2) polymorphisms and risk for alcohol dependence. J. Psychiatr. Res. 42, 184–191. doi: 10.1016/j.jpsychires.2006.11.006, PMID: 17207817

[ref174] Stephens D. N. Pistovcakova J. Worthing L. Atack J. R. Dawson G. R. (2005). Role of GABAA alpha5-containing receptors in ethanol reward: the effects of targeted gene deletion, and a selective inverse agonist. Eur. J. Pharmacol. 526, 240–250. doi: 10.1016/j.ejphar.2005.09.031, PMID: 16253225

[ref175] Stokes M. Abdijadid S. (2022). Disulfiram in StatPearls. Treasure Island, FL: StatPearls Publishing Copyright.

[ref176] Tanabe J. Yamamoto D. J. Sutton B. Brown M. S. Hoffman P. L.. (2019). Effects of alcohol and acetate on cerebral blood flow: A pilot study. Alcohol. Clin. Exp. Res. 43, 2070–2078. doi: 10.1111/acer.14173, PMID: 31386214 PMC7066986

[ref177] Tarren J. Shariff M. Holgate J. Bartlett S. E. (2016). “Chapter 38 – effects of alcohol on nicotinic acetylcholine receptors and impact on addiction” in Neuropathology of drug addictions and substance misuse. ed. Preedy V. R. (San Diego: Academic Press), 411–419.

[ref178] Tateno T. Robinson H. P. C. (2011). The mechanism of ethanol action on midbrain dopaminergic neuron firing: a dynamic-clamp study of the role of IH and GABAergic synaptic integration. J. Neurophysiol. 106, 1901–1922. doi: 10.1152/jn.00162.2011, PMID: 21697445

[ref179] Tehrani M. H. Barnes E. M. (1997). Sequestration of γ-aminobutyric acidA receptors on clathrin-coated vesicles during chronic benzodiazepine administration *in vivo*. J. Pharmacol. Exp. Ther. 283, 384–390. 9336347

[ref180] Terunuma M. (2018). Diversity of structure and function of GABA(B) receptors: a complexity of GABA(B)-mediated signaling. Proc. Jpn. Acad. Ser. B Phys. Biol. Sci. 94, 390–411. doi: 10.2183/pjab.94.026, PMID: 30541966 PMC6374141

[ref181] Thaysen-Petersen D. Hammerum S. K. Vissing A.-C. Arnfred B. T. Nordahl R.. (2023). Virtual reality-assisted cognitive behavioural therapy for outpatients with alcohol use disorder (CRAVR): a protocol for a randomised controlled trial. BMJ Open 13:e068658. doi: 10.1136/bmjopen-2022-068658, PMID: 36990475 PMC10069573

[ref182] Tizabi Y. Bai L. Copeland R. L. Taylor R. E. (2007). Combined effects of systemic alcohol and nicotine on dopamine release in the nucleus accumbens shell. Alcohol Alcohol. 42, 413–416. doi: 10.1093/alcalc/agm057, PMID: 17686828

[ref183] Trantham-Davidson H. Chandler L. (2015). Alcohol-induced alterations in dopamine modulation of prefrontal activity. Alcohol 49, 773–779. doi: 10.1016/j.alcohol.2015.09.001 26558348 PMC4691370

[ref184] Trudell J. R. Messing R. O. Mayfield J. Harris R. A. (2014). Alcohol dependence: molecular and behavioral evidence. Trends Pharmacol. Sci. 35, 317–323. doi: 10.1016/j.tips.2014.04.009, PMID: 24865944 PMC4089033

[ref185] Turton S. Myers J. F. M. Mick I. Colasanti A. Venkataraman A.. (2020). Blunted endogenous opioid release following an oral dexamphetamine challenge in abstinent alcohol-dependent individuals. Mol. Psychiatry 25, 1749–1758. doi: 10.1038/s41380-018-0107-4, PMID: 29942043 PMC6169731

[ref186] Valenzuela C. F. (1997). Alcohol and neurotransmitter interactions. Alcohol Health Res. World 21, 144–148. PMID: 15704351 PMC6826822

[ref187] Valenzuela C. F. Jotty K. (2015). Mini-review: effects of ethanol on GABAA receptor-mediated neurotransmission in the cerebellar cortex—recent advances. Cerebellum 14, 438–446. doi: 10.1007/s12311-014-0639-3, PMID: 25575727

[ref188] Vengeliene V. Bachteler D. Danysz W. Spanagel R. (2005). The role of the NMDA receptor in alcohol relapse: a pharmacological mapping study using the alcohol deprivation effect. Neuropharmacology 48, 822–829. doi: 10.1016/j.neuropharm.2005.01.002, PMID: 15829254

[ref189] Volkow N. D. Wang G.-J. Tomasi D. Baler R. D. (2013). Unbalanced neuronal circuits in addiction. Curr. Opin. Neurobiol. 23, 639–648. doi: 10.1016/j.conb.2013.01.002, PMID: 23434063 PMC3717294

[ref190] Walzer M. Marek G. J. Wu R. Nagata M. Han D. (2020). Single-and multiple-dose safety, tolerability, and pharmacokinetic profiles of ASP8062: results from 2 phase 1 studies. Clin. Pharmacol. Drug Dev. 9, 297–306. doi: 10.1002/cpdd.766, PMID: 31926000

[ref191] Wenzel R. G. Schwarz K. Padiyara R. S. (2006). Topiramate for migraine prevention. Pharmacotherapy 26, 375–387. doi: 10.1592/phco.26.3.375, PMID: 16503717

[ref192] Weresch J. Kirkwood J. Korownyk C. S. (2021). Gabapentin for alcohol use disorder. Can. Fam. Physician 67:269. doi: 10.46747/cfp.6704269, PMID: 33853915 PMC8324154

[ref193] Werner D. F. Porcu P. Boyd K. N. O'Buckley T. K. Carter J. M.. (2016). Ethanol-induced GABAA receptor alpha4 subunit plasticity involves phosphorylation and neuroactive steroids. Mol. Cell. Neurosci. 72, 1–8. doi: 10.1016/j.mcn.2016.01.002, PMID: 26805653 PMC4932829

[ref194] Wetherill R. R. Fromme K. (2016). Alcohol-induced blackouts: A review of recent clinical research with practical implications and recommendations for future studies. Alcohol. Clin. Exp. Res. 40, 922–935. doi: 10.1111/acer.13051, PMID: 27060868 PMC4844761

[ref195] Wetherill R. R. Spilka N. Jagannathan K. Morris P. Romer D.. (2021). Effects of topiramate on neural responses to alcohol cues in treatment-seeking individuals with alcohol use disorder: preliminary findings from a randomized, placebo-controlled trial. Neuropsychopharmacology 46, 1414–1420. doi: 10.1038/s41386-021-00968-w, PMID: 33558678 PMC8208990

[ref196] WHO. (2021). Global strategy to reduce harmful use of alcohol. Geneva: WHO

[ref197] WHO-ICD11. (2022). International classification of diseases: Eleventh revision (ICD-11). Geneva: WHO

[ref198] Wilson D. F. Matschinsky F. M. (2020). Ethanol metabolism: the good, the bad, and the ugly. Med. Hypotheses 140:109638. doi: 10.1016/j.mehy.2020.109638, PMID: 32113062

[ref199] World Health Organization. (2000). International guide for monitoring alcohol consumption and related harm. Geneva: World Health Organization

[ref200] Yang W. Singla R. Maheshwari O. Fontaine C. J. Gil-Mohapel J. (2022). Alcohol use disorder: neurobiology and Therapeutics. Biomedicine 10:1192. doi: 10.3390/biomedicines10051192, PMID: 35625928 PMC9139063

[ref201] Zangen A. Moshe H. Martinez D. Barnea-Ygael N. Vapnik T.. (2021). Repetitive transcranial magnetic stimulation for smoking cessation: a pivotal multicenter double-blind randomized controlled trial. World Psychiatry 20, 397–404. doi: 10.1002/wps.20905, PMID: 34505368 PMC8429333

[ref202] Zaporowska-Stachowiak I. Szymański K. Oduah M.-T. Stachowiak-Szymczak K. Łuczak J. Sopata M. (2019). Midazolam: safety of use in palliative care: A systematic critical review. Biomed. Pharmacother. 114:108838. doi: 10.1016/j.biopha.2019.108838, PMID: 30981104

[ref203] Zhao J. Stockwell T. Naimi T. Churchill S. Clay J. Sherk A. (2023). Association between daily alcohol intake and risk of all-cause mortality: A systematic review and meta-analyses. JAMA Netw. Open 6:e236185. doi: 10.1001/jamanetworkopen.2023.6185, PMID: 37000449 PMC10066463

[ref204] Ziemann U. Reis J. Schwenkreis P. Rosanova M. Strafella A.. (2015). TMS and drugs revisited 2014. Clin. Neurophysiol. 126, 1847–1868. doi: 10.1016/j.clinph.2014.08.028, PMID: 25534482

[ref205] Zorumski C. F. Paul S. M. Izumi Y. Covey D. F. Mennerick S. (2013). Neurosteroids, stress and depression: potential therapeutic opportunities. Neurosci. Biobehav. Rev. 37, 109–122. doi: 10.1016/j.neubiorev.2012.10.005, PMID: 23085210 PMC3591791

